# Mitochondrial genomic variation drives differential nuclear gene expression in discrete regions of Drosophila gene and protein interaction networks

**DOI:** 10.1186/s12864-019-6061-y

**Published:** 2019-09-02

**Authors:** Jim A. Mossman, Leann M. Biancani, David M. Rand

**Affiliations:** 1 Department of Ecology and Evolutionary Biology, Box G, Brown University, Providence, RI 02912 USA; 2 Present Address: Department of Biology, University of Maryland, College Park, MD 20742 USA

**Keywords:** Mitonuclear, mtDNA, Haplotype, Gene expression, Systems biology

## Abstract

**Background:**

Mitochondria perform many key roles in their eukaryotic hosts, from integrating signaling pathways through to modulating whole organism phenotypes. The > 1 billion years of nuclear and mitochondrial gene co-evolution has necessitated coordinated expression of gene products from both genomes that maintain mitochondrial, and more generally, eukaryotic cellular function. How mitochondrial DNA (mtDNA) variation modifies host fitness has proved a challenging question but has profound implications for evolutionary and medical genetics. In Drosophila, we have previously shown that recently diverged mtDNA haplotypes within-species can have more impact on organismal phenotypes than older, deeply diverged haplotypes from different species. Here, we tested the effects of mtDNA haplotype variation on gene expression in Drosophila under standardized conditions. Using the Drosophila Genetic Reference Panel (DGRP), we constructed a panel of mitonuclear genotypes that consists of factorial variation in nuclear and mtDNA genomes, with mtDNAs originating in *D. melanogaster* (2x haplotypes) and *D. simulans* (2x haplotypes).

**Results:**

We show that mtDNA haplotype variation unequivocally alters nuclear gene expression in both females and males, and mitonuclear interactions are pervasive modifying factors for gene expression. There was appreciable overlap between the sexes for mtDNA-sensitive genes, and considerable transcriptional variation attributed to particular mtDNA contrasts. These genes are generally found in low-connectivity gene co-expression networks, occur in gene clusters along chromosomes, are often flanked by non-coding RNA, and are under-represented among housekeeping genes. Finally, we identify the *giant* (*gt*) transcription factor motif as a putative regulatory sequence associated with mtDNA-sensitive genes.

**Conclusions:**

There are predictive conditions for nuclear genes that are influenced by mtDNA variation.

**Electronic supplementary material:**

The online version of this article (10.1186/s12864-019-6061-y) contains supplementary material, which is available to authorized users.

## Background

Mitochondria are master regulators of cellular function, cell death, signaling and a host of metabolic processes including ATP production and fatty acid oxidation [1, 2]. As major mediators of cellular processes, dysfunction of mitochondria has been associated with a large number of pathologies [3], which can have a strong genetic basis [4–6]. The genetics of mitochondrial disease and mitochondrial dysfunction more generally is complex because the mitochondrion is encoded by two distinct genomes; the nuclear genome (nDNA) encodes > 1000 gene products that function in the mitochondrion, and the mitochondrial genome (mtDNA) contains 13 protein coding genes, 22 transfer RNAs and two ribosomal RNAs that are expressed within the organelle. Greater than one billion years of nuclear and mtDNA gene coevolution [7] has necessitated the coordinated expression of genes on each genome to precisely control protein products in the two-genome-encoded electron transport chain (ETC) – a genetic model for gene-gene and protein-protein interactions (GGI and PPI, respectively) [8]. The protein products of both genomes are required for efficient mitochondrial biogenesis, and mutations in both nDNA and mtDNA in isolation or in combination can cause deleterious phenotypic variation [6, 9, 10].

MtDNAs accumulate mutations at a high rate and these may be in the form of SNPs, or small and large scale deletions [11]. How these haplotype variants, deleterious somatic point mutations, and large scale deletions affect phenotypes has been an active research area for the last 30 years [12, 13] and is motivating promising new approaches to prevent and treat inherited mtDNA-associated diseases in humans [14–17] .

Mitochondrial replacement therapies are among the most promising of these therapies, but they face the challenge of identifying and circumventing unfavorable (negative) interactions between mtDNA and nDNA. We refer to these types of gene x gene interactions as mitonuclear epistases (G x G). Mitonuclear epistases are pervasive in model organism research [18–24] and are largely unpredictable. More generally, epistatic interactions between nuclear genes are presumed to explain a significant amount of the ‘missing heritability’ in complex traits [25, 26] and diseases [27, 28], therefore any pharmacogenomic or personalized medicine approach to disease management will require precise knowledge of how genes interact with their genetic and physical environments to accurately predict efficacy and safety.

We have successfully mimicked a mitochondrial disease in the fruit fly, *Drosophila melanogaster,* using mitonuclear introgression of isogenic nuclear backgrounds and variable mitochondrial haplotypes in a phylogenetic context [19, 29]. Importantly, we have shown that the amount of genetic distance between mtDNA haplotypes (numbers of synonymous or non-synonymous mutations) is a poor predictor of whole organism phenotypes [18, 20]. For example, if mutation load/sequence divergence per se is a predictor of phenotypic divergence, one would expect the organisms harboring the most dissimilar mtDNA haplotypes to demonstrate the most divergent phenotypic variation. In the majority of our studies we have failed to observe this simple expectation of genome co-adaptation/co-evolution, partly because mtDNA haplotypes exert their effects in a context-dependent manner [20, 21, 30]. That is, haplotype substitutions behave differently depending on the isogenic nuclear genome they are paired with (G x G sensitive), or the abiotic environment they are placed in (G x G x E effect) [20, 22, 30–33]. We have observed mitonuclear genotypes that do follow this simple coadaptation rule, but these represent only a minority of the tested mitonuclear epistatic combinations [20, 34]. The questions therefore are why are some nuclear genetic backgrounds more sensitive to mtDNA variation (ΔmtDNA) than others? And could we use the variable penetrance of mtDNA variation on mitochondrial disease [35] or phenotypic expression to identify core regions of the interactome [36] that are sensitive to ΔmtDNA? This might help turn a hitherto measurement exercise into a predictive model.

Since nuclear genetic backgrounds can exert a large influence on the sensitivity to mtDNA variation, and the effects do not repeatedly follow the simple expectations of co-adaptation, at least in Drosophila, we postulate that underlying higher-order genetic and protein interaction networks are central modifiers of the sensitivity to ΔmtDNA. The lack of uniform ΔmtDNA effects across the majority of nuclear genetic backgrounds [22, 37] (see also [38]) suggests that core networks of genes that play fundamental, or housekeeping roles, are not exposed to ΔmtDNA effects. We therefore wanted to ask how mtDNA variation alters gene expression in the context of GGI and PPIs.

To test this hypothesis we used a subset of a previously constructed panel of *D. melanogaster* mitonuclear genotypes whose nuclear variation originates in the Drosophila Genetic Reference Panel (DGRP). The nuclear backgrounds used were *DGRP-315* and *DGRP-820* since these revealed a sensitivity to mtDNA for the whole organism phenotype: egg-to-adult development time [20]. Using these two nDNA backgrounds, we tested whether mtDNA influenced nuclear gene expression (a) within a nuclear background (haplotype effects) (4 mtDNA × 1 nDNA (*DGRP-315*)), and (b) across nuclear genotypes (G x G) (2 mtDNA × 2 nDNA (*DGRP-315* and *DGRP-820*)). We found significant mtDNA ‘haplotype’ and mtDNA ‘species’ effects that are enriched in low-connectivity regions of GGI networks, suggesting ΔmtDNA does not influence hubs, or highly connected network regions. We also observed non-random clustering of highly related or tandem duplicated genes that were also sensitive to ΔmtDNA. Furthermore, mtDNA effects are under-enriched in housekeeping genes. We further show that these mtDNA-sensitive genes, while evident in low-connectivity regions of GGI and PPI networks, have a strong signal for the transcription factor binding motif associated with *giant* (*gt*). The strong association between transcription factor binding sites may provide a systems-wide explanation of why only certain regions of the GGI and PPI networks are sensitive to mtDNA variation, providing a gene regulation component of mtDNA effects.

## Results

### mtDNA effects on nuclear gene expression are numerous and roughly equal in each sex

**Fig. 1:**
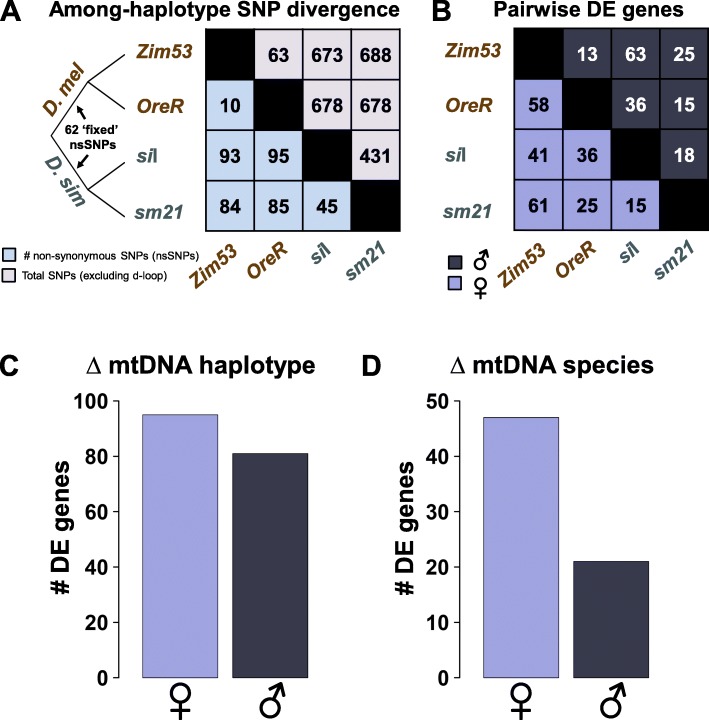
Experiment design and *DGRP-315* differentially expressed (DE) gene numbers. The cartoon phylogeny in (**a**) describes the two *D. melanogaster* (*D. mel*) haplotypes, and the two *D. simulans* (*D. sim*) haplotypes. Branch lengths are not proportional to the degree of divergence. Numbers of non-synonymous SNPs among the haplotypes are reported in the bottom section of (**a**) in light blue. Numbers of total genome-wide SNP substitutions (excluding the AT-rich control region) are shown in the top part of the figure in pink. Pairwise contrasts: the numbers of DE genes (FDR < 0.05) in each pairwise haplotype contrast are shown in (**b**). Female contrasts are shown in violet (bottom of plot). Male contrasts are shown in the top part in dark blue. Numbers of DE genes (FDR < 0.05) determined by analysis of deviance-type analyses are shown for ΔmtDNA ‘haplotype’ (**c**) and ΔmtDNA ‘species’ (**d**)

Our experimental design allowed examination of between-mtDNA haplotype contrasts at the individual haplotype and between-species levels. The phylogenetic framework for mtDNA contrasts is shown in Fig. 1a. The dataset for the *DGRP-315* background consisted of four mtDNA haplotypes that are differentiated by up to 95 amino acid polymorphisms in their protein-coding sequences and up to 688 genome-wide SNPs across all mtDNA features except the AT-rich control region [18, 39]. We first asked whether there was a correlation between the numbers of pairwise amino acid substitutions in the protein coding region of the mtDNA and the numbers of differentially expressed (hereafter, DE) genes with FDR < 0.05 in the pairwise gene expression contrasts (Fig. 1b). We found no evidence that mtDNA amino acid divergence was associated with the number of DE genes in the combined female and male datasets (Pearson’s *r* = 0.22, *p* = 0.49), the female dataset alone (Pearson’s *r* = 0.59, *p* = 0.21), and the male dataset (Pearson’s *r* = − 0.15, *p* = 0.77). We next tested whether the numbers of total substitutions across all features (excluding the AT-rich control region) were correlated with DE genes. We found qualitatively the same result of no correlation between molecular divergence and numbers of DE genes: females and males combined (Pearson’s *r* = 0.13, *p* = 0.68), females alone (Pearson’s *r* = 0.49, *p* = 0.32), and males alone (Pearson’s *r* = − 0.23, *p* = 0.66). These results suggest that our earlier studies’ evidence of no consistent molecular distance effects at the expression of whole organism phenotypes is recapitulated with expression of transcripts, at least to the level of divergence between the mtDNAs of *D. melanogaster* and *D. simulans*.

We next performed an analysis of deviance (ANODEV)-type test [40, 41] across all four mtDNA haplotypes in each sex. In this ΔmtDNA ‘haplotype’ test all female haplotypes were simultaneously compared and all male haplotypes were simultaneously compared in two independent separate-sex analyses. We first created a matrix of all six possible independent contrasts in *edgeR* (e.g. *Zim53-OreR*, *Zim53-si*I, *Zim52-sm21*, *OreR-si*I, *OreR-sm21*, *si*I*-sm21*). To identify genes that were DE between the four haplotypes, we performed generalized linear model likelihood ratio tests on the model fit using the glmLRT function and tag-wise dispersion estimates, as implemented in *edgeR*. We found the numbers of genes that were differentially expressed within the *DGRP-315* nuclear background to be roughly equal in females (FDR < 0.05, *n* = 95 genes) and males (FDR < 0.05, *n* = 81 genes) (Fig. 1c). A similar result was found for mtDNA ‘species’ effects in which a larger number of genes were DE in females (FDR < 0.05, *n* = 47) than males (FDR < 0.05, *n* = 21) (Fig. 1d). For these ΔmtDNA ‘species’ analyses, the RNAseq count data representing the *D. mel* haplotypes (*Zim53* and *OreR*) were grouped together and contrasted against the *D. sim* haplotypes (*si*I and *sm21*), which were also combined. The smaller numbers of genes found at the ‘species’ level is consistent with the overall lack of molecular distance effect, but it is important to note that these effects cannot be delineated with a small number of haplotypes within each species. For example, a large individual within-species mtDNA ‘haplotype’ effect can reveal itself as a ‘species’ effect if the variance between replicates of the other haplotypes is low.

We next wanted to test whether broad scale mitonuclear epistasis (interactions between the complete mtDNA and complete nDNA genomes, and not specific SNP interactions) were present in a sub-set of these genotypes. The genotypes we used in this test were *Zim53;DGRP-315*, *sm21;DGRP-315*, *Zim53;DGRP-820*, and *sm21;DGRP-820*. The epistasis model was the haplotype difference within *DGRP-315* nuclear background contrasted with the haplotype difference in the DGRP-820 nuclear background (e.g. (*Zim53;DGRP-315- sm21;DGRP-315*) *–* (*Zim53;DGRP-820* - *sm21;DGRP-820*)). The numbers of genes that were influenced by broad scale mitonuclear epistasis was much larger in females (FDR < 0.05, *n* = 606 genes) than males (FDR < 0.05, *n* = 18), suggesting the *Zim53*-*sm21* mtDNA haplotype contrast was variable across *DGRP-315* and the alternative *DGRP-820* nuclear backgrounds (see Methods for details), and that effect was more pronounced in females. In the remainder of this article we refer to the deviance analysis as ΔmtDNA ‘haplotype’, and the species-level analysis as ΔmtDNA ‘species’. The epistasis contrast is referred to as ‘mitonuclear variation’ and ‘G x G’.

There was measurable overlap between the sexes for those genes DE by ΔmtDNA ‘haplotype’. The list of the FDR < 0.05 DE genes are shown in Additional file 1: Table S1, and contains genome coordinates and gene IDS of the private and overlapping (shared) genes. There was a strong representation of ionotropic receptors and cuticular proteins in the female-specific list, and *Turandot* and seminal proteins in males. The shared list was dominated by mitochondrial DNA genes and ionotropic receptors (*Ir67c* and *Ir93a*), along with *shadow*, *actin 88F*, and odorant receptors (*Or85F* and *Or67a*). Gene ontology analysis of the female DE genes revealed the most enriched *process* term was ‘chitin-based cuticle development’ (GO:0040003; FDR q-value [42]=2.56e-04), while the most enriched term in males was ‘cellular response to UV’ (GO:0034644), although this was not significant after multiple test correction (Benjamini and Hochberg [42] q = 0.39). Of the 20 most significantly DE genes in each sex, there was an 11 gene overlap. Six of these genes were mtDNA-encoded, and the remaining genes were: *shadow* (a mitochondrial-localized cytochrome P450 involved in ecdysteroid biosynthesis), *Ionotrophic receptor 93a* (a glutamate receptor), *CG33465* (a serine protease), *Ionotropic receptor 67c* (a membrane bound ligand gated ion channel protein), and *Odorant receptor 85f* (a transmembrane chemoreceptor that mediates a response to volatile chemicals).

There was a smaller representation of significant ΔmtDNA ‘species’ genes (Fig. 1d) and small overlap between males and females for the genes that are DE (FDR < 0.05; Additional file 2: Table S2). Among the genes that were consistently DE across the sexes and not mtDNA-encoded were: *CG40211* (formally mapped to heterochromatin region of 2R [43])*, lethal (2) giant larvae, Ionotropic receptor 47a, CG33465, Ionotropic receptor 67c, CG1750*, *shadow*, and *Ionotropic receptor 93a*.

### MtDNA-sensitive genes are physically clustered throughout the genome

**Fig. 2:**
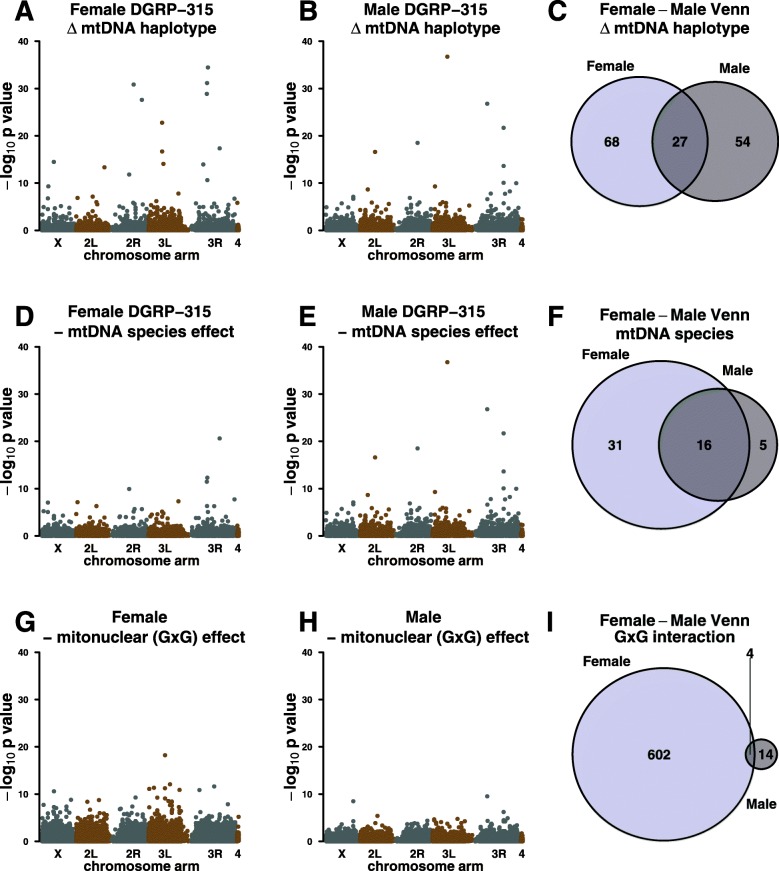
Physical location and ID overlap between the sexes of DE genes in the *DGRP-315* nuclear background. Physical chromosome locations are shown on the abscissa of the Manhattan plots (**a**, **b**, **d**, **e**, **g**, **h**) and the significance (−log_10_ *p*-value) is shown on the ordinal scale. Each datum represents an individual transcript. Female ΔmtDNA ‘haplotype’, ΔmtDNA ‘species’, and G x G ‘broadscale epistasis’ distributions are shown in **a**, **d** and **g**, respectively. Male ΔmtDNA ‘haplotype’, ΔmtDNA ‘species’, and G x G ‘broadscale epistasis’ distributions are shown in **b**, **e**, and **h**, respectively. Between-sex Venn intersections of DE genes (FDR < 0.05) and shown for ΔmtDNA ‘haplotype’, ΔmtDNA ‘species’, and G x G ‘broadscale epistasis’ in **c**, **f**, and **i**, respectively. Broadscale epistasis represents the totality of interactions between the complete nuclear genomes and the complete mtDNA genomes. In other words, these are not specific SNP interactions, but whole scale genome interactions. Broadscale mitonuclear interaction genes are estimated as the (*Zim53-sm21*) mtDNA contrast in the DGRP-315 background contrasted against the (*Zim53-sm21*) contrast in the DGRP-820 background

To examine whether there are physical regions of the genome that show enrichment (clustering) of mtDNA-sensitive genes we plotted a Manhattan-style figure of the log_10_ *p*-value against the linearized genome coordinates (Fig. 2). We then formally analyzed the distribution of genes along the chromosomes using Cluster Locator (http://clusterlocator.bnd.edu.uy/) [44] and the *D. melanogaster* reference genome (Flybase Release 6.17) with a Max-gap = 5 parameter. Two-sided Kolmogorov-Smirnov tests were used to determine if the genes entered in the list were uniformly distributed along the chromosome arms and whether the numbers of realized clusters is different to 1000 randomly generated gene lists. We chose the Top 200 *p*-value ranked genes as the ‘test set’ in each contrast, complimentary to the contrasts shown in Fig. 2: ΔmtDNA ‘haplotype’ variation (Fig. 2a, b); ΔmtDNA ‘species’ variation (Fig. 2d, e); mitonuclear (G x G) variation (Fig. 2g, h) in both females and males, respectively. The top 200 genes represent the top 1.4% of genes as ranked by their p-value significance. In all six analyses, the top 200 genes all had an un-adjusted *p* < 0.05 but the top 200 ‘test set’ was used to maintain an equivalent number of genes in each cluster analysis.

**Fig. 3:**
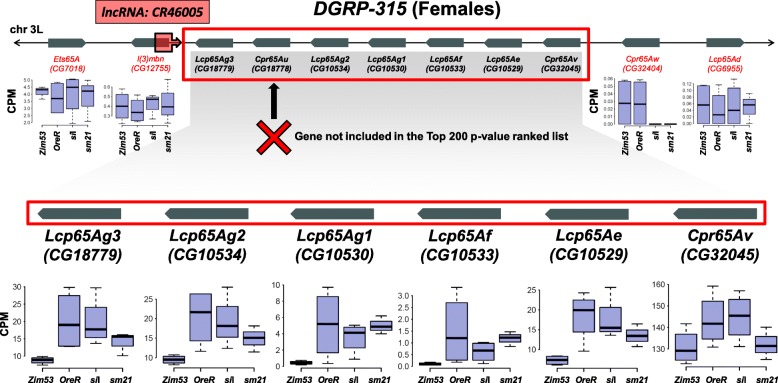
Differentially expressed genes statistically cluster within chromosomes. Shown is an example of a female gene cluster demonstrating consistent norms of reaction across contiguous genes on the 3R chromosome arm. The cluster is flanked by a non-coding RNA, a feature we identified across multiple clusters. The run of significant and contiguous genes is bounded by non-significant genes. CPM = counts per million, a measure of relative transcript expression

In all gene lists, we identified gene clusters that are not randomly distributed across chromosome arms and which are sensitive to one or more forms of mtDNA variation (Table 1). For example, genes that are sensitive to ΔmtDNA haplotype are often clustered and adjacent e.g. a cluster of genes in the chitin-related *Tweedle* gene family coupled with a cluster of chitin protein genes, both on chromosome 3R in females (Fig. 3). Likewise, in males, significant *Turandot* genes on the 3R chromosome arm are clustered.

**Table 1:** MtDNA-sensitive genes are more likely to be clustered than randomly distributed. Results of Cluster Locator [44] analyses are shown, along with D (a uniform distribution test statistic) and the p-value of the distribution. The proportion of genes that are statistically clustered and the respective p-value are shown for each contrast type. The bottom part of the table includes the size distribution of clusters in each analysis type. The integer represents the number of clusters of a given size between 2 and 7 genes. Obviously, the portion of the chromosome can include many more genes as the gap penalty was set at five for all analyses (e.g. there can be up to five genes between the ‘clustered’ genes and a cluster size of 7 represents a potential window size of up to 32 genes)

			Chromosome arm											
			2 L		2R		3 L		3R		X			
Sex	Contrast	# Clusters	*D*	*P*	*D*	*P*	*D*	*P*	*D*	*P*	*D*	*P*	% genes clustered	*P*-value
F	*Δ mtDNA haplotype*	27	0.21	0.59	0.18	0.36	0.08	0.82	0.13	0.50	0.21	0.20	43.53	**< 1.00e-10**
F	*Δ mtDNA species*	18	0.14	0.59	0.15	0.41	0.16	0.34	0.16	0.17	0.25	0.06	24.56	**8.12E-04**
F	*G x G effect*	27	0.15	0.46	0.25	**0.04**	0.15	0.10	0.10	0.83	0.19	0.15	38.02	**< 1.00e-10**
M	*Δ mtDNA haplotype*	21	0.18	0.18	0.21	0.13	0.12	0.62	0.09	0.77	0.20	0.42	28.25	**6.86E-05**
M	*Δ mtDNA species*	19	0.12	0.89	0.25	**0.01**	0.20	0.07	0.09	0.79	0.26	0.27	25.15	**5.88E-04**
M	*G x G effect*	20	0.10	0.96	0.09	0.87	0.16	0.48	0.14	0.23	0.17	0.60	27.88	**2.75E-05**
Cluster size distribution (number of genes in cluster, gaps = 5)														
		2	3	4	5	6	7							
F	*Δ mtDNA haplotype*	18	3	2	3	1								
F	*Δ mtDNA species*	15	1	1	1									
F	*G x G effect*	19	3	2	1	1	1							
M	*Δ mtDNA haplotype*	17	1	2	1									
M	*Δ mtDNA species*	15	3	1										
M	*G x G effect*	15	4	1										

*p*-values in bold are significant at α=0.05

Large, non-random gene clusters tended to be tandem repeats of duplicated genes and these are often flanked by long and short non-coding RNAs and antisense RNAs, and associated with transcription factor binding site hot spots. These non-random gene clusters are not surprising since genes that are physically linked by necessity of their functions or spatial-temporal specificity are likely to be under similar co-expression / co-suppression patterns [45]. Indeed, we find that mtDNA-sensitive genes that are physically clustered show similar norms of reaction across the mtDNA haplotypes, and these clusters therefore suggest shared regulatory elements (Fig. 3). Females generally showed a larger number of clusters in the three contrast types (Table 1), which is expected given females also show slightly greater numbers of significant genes. Crucially though, in both sexes and across all three contrast types the proportions of genes that are found in clusters are greater than would be expected by chance alone (*p* <  1e-10; Table 1). This finding suggests that the mtDNA impact on nuclear genes involves higher-order regulatory interactions including co-expressed genes that are functionally, and in many cases, physically linked. The significant physical clustering in the most mtDNA-sensitive transcripts is further supported by re-analysis of data from a previous mitonuclear gene expression study [21, 30]. In both females and males the signature of statistical physical clustering is qualitatively consistent if a much smaller list of genes passing FDR < 0.05 is used (Additional file 6: Table S6). In some cases the signal remains with samples of the top 200–400 *p*-value ranked genes, but does erode rapidly with descending position in sequential sets of 200 genes in such a list (Additional file 9: Figure S3).

There was considerable overlap between females and males for the genes that were sensitive to the above forms of variation. Using a FDR < 0.05 cut-off, 27 genes were shared between the sexes for ΔmtDNA ‘haplotype’ variation (Fig. 2c; Additional file 1: Table S1), 16 genes were shared between the sexes for ΔmtDNA ‘species’ variation (Fig. 2f; Additional file 2: Table S2), and four were shared between the sexes for mitonuclear (G x G) variation (Fig. 2i).

### Gene networks with high mtDNA-sensitive gene representation are poorly connected

So far, we have shown the architecture of mtDNA-sensitive genes is not random with respect to genome physical location and mean expression level. To better understand if the non-random associations were related to the underlying gene co-expression networks, we mapped significant mtDNA-sensitive genes onto a de novo Drosophila Genetics Reference Panel (DGRP) gene co-expression network constructed using a Weighted Gene Co-expression Network Analysis (WGCNA) (see Methods for details). The WGCNA network we produced from the 185-genotype DGRP whole transcriptome resource [46] contained 16 proper modules of statistically co-expressed genes, and one improper module containing genes that cannot be statistically grouped with a co-expression gene set (e.g. those genes in proper modules) [47]. By far the largest proportion of the genes that were sensitive to mtDNA variation in both males and females were associated with the improper module with extremely low or zero connectivity, suggesting mtDNA sensitive genes are underrepresented in well-connected modules, even though we identified local co-regulation signatures on small numbers of clustered genes (above; Fig. 3). Approximately 65% of ΔmtDNA ‘haplotype’-sensitive genes (*P* < 0.05 threshold) in females are found in the improper module. The zero-linkage improper module represents ~ 42% of the total genes that were analyzed in the network, resulting in an overall and significant 1.49 x enrichment of mtDNA-sensitive genes (Hypergeometric test: *p* = 3.77e-22) in that module. The same qualitative effect was observed if we used a strict significance cut-off of FDR < 0.05 (enrichment: 1.45 x expected, Hypergeometric test: *p* < 0.001). We observed the same effect in males for *P* < 0.05 threshold (1.50 x enrichment, Hypergeometric test: *p* = 3.30e-34) and FDR < 0.05 (1.73 x expected, Hypergeometric test, *p* = 1.69e-08). Taken together, we find that the poorly-connected module with no signature of gene co-expression is enriched for genes that are sensitive to mtDNA variation in both sexes.

Our main motivation for using the complete DGRP collection to construct our co-expression gene network was partly to capture the network topology that is evident across a large number of genotypes (185 DGRP lines), but mainly because we did not want the network topology to be constrained or influenced by our experimental design. In order to confirm that our mtDNA-sensitive gene enrichment in poorly connected improper modules was repeatable in an independently constructed network, we also performed WGCNA on the RNA-seq reads we generated in the current investigation. We identified 31 modules of co-expressed genes and one improper module of low/zero connectivity genes. In our second de novo network, the improper (low/zero connectivity) module contained 2802 genes out of a total 13,738 (~ 20.4%), yet it contained 69/95 (72.6%) of the significant ΔmtDNA haplotype-sensitive genes in females (3.5 x expected, Hypergeometric test: *p* = 3.90e-28), and 57/81 (70.4%) of the significant ΔmtDNA haplotype genes in males (3.4 x expected, Hypergeometric test: *p* = 3.31e-22). In both of our constructed networks there were several modules that contained mtDNA-sensitive genes (Additional file 7: Figure S1) however no individual module was statistically overrepresented, or enriched.

### MtDNA contrasts provide unique signatures of enrichment in protein-protein interaction networks

We have previously documented genotype-specific responses to mitonuclear variation whereby different nuclear backgrounds demonstrate almost unique transcript responses to mtDNA variation in Drosophila [21, 30]. We have further demonstrated that gene-gene module connectivity is strongly associated with the sensitivity of a gene to mtDNA variation (see above). We next wanted to test the hypothesis that the genes that are induced or suppressed when placed in an alternative mtDNA environment, are connected through functional pathways, and/or have some underlying co-regulatory signature.

**Fig. 4:**
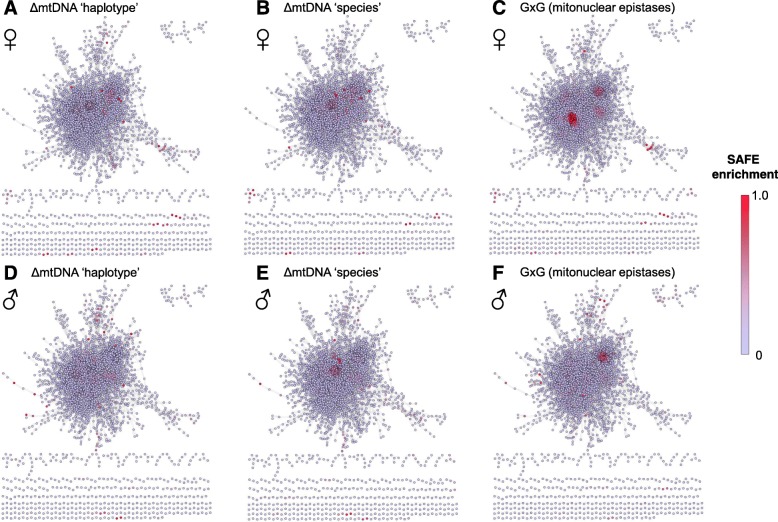
Mapping DE gene enrichments using SAFE and a PPI network analysis. The network topology used in these analyses is based on the network of [49]. The network topology is identical in **a**-**f**. Highly connected genes are present at the center of the network. Poorly connected (~ 2 to 20 gene) sub networks are shown at the bottom and top right of each figure. Enriched regions of DE genes in the PPI network are displayed as a heat component. Red is enriched, violet is not enriched. Contrasts are as follows: ΔmtDNA ‘haplotype’ (**a**, **d**), ΔmtDNA ‘species’ (**b**, **e**), and G x G ‘broadscale epistasis’ (**c**, **f**). Female analyses are shown in A, B, and C, male analyses are shown in D, E, and F Contrasts show both conserved and private regions of enrichment across contrasts. The six remaining haplotype pairwise comparisons (for females as an example) are shown in Additional file 8: Figure S2

To test this we used systematic functional annotation and visualization of biological networks (SAFE) [48], along with network analysis to compare and map enriched network regions in an established protein-protein interaction (PPI) network [49]. In this analysis, each node of the network (protein-coding gene) has an attribute score: the log_10_ *p*-value of an mtDNA contrast of interest. The sum of the attribute scores (−log_10_ *p*-values) in the focal gene’s local interaction neighborhood is then compared to a random expectation model and a p-value is calculated. The degree of neighborhood enrichment of high or low attribute values is represented by a heat component and visually represented by color intensity. Figure 4 shows the SAFE enrichments of a subset (ΔmtDNA haplotype (Fig. 4a & d), ΔmtDNA species (Fig. 4b & e), and GXG (Fig. 4c & f)) of all mtDNA contrasts in females (Fig. 4a, b & c) and males (Fig. 4d, e & f). Across the nine analysis types (Additional file 8: Figure S2), there are regions of the PPI network that are consistently enriched for proteins whose genes are highly differentially expressed and in the same way there are regions that are unique in each analysis. Consistent with the GGI analysis, we find poorly connected regions of the PPI network to have proportionally more mtDNA-sensitive genes. The main difference between the GGI and PPI networks is that the PPI network is composed of known and validated interactions. As a result, negligible strength protein-protein interactions, the equivalent of zero/low-connectivity improper GGI modules, are not present in the PPI network. Nevertheless, low-connectivity regions of the PPI network are over represented, suggesting central hubs of the PPI are not major sources of mtDNA-sensitive genes (Fig. 4).

We formally tested the neighborhood connectivity [50] of significant and non-significant genes using NetworkAnalyzer [51] and found the ΔmtDNA ‘haplotype’ DE genes (top 200 ranked by *p*-value) had a significantly smaller neighborhood connectivity than non-significant genes in both sexes. In females the average neighborhood sizes of significant (high ranking) and non-significant (remaining) genes were 18.74 and 26.18, respectively (Welch’s Two sample t-test: t = 4.734, df = 245.67, *p* = 3.721e-06). In males the high ranking genes were from average neighborhood sizes of 14.97 (significant genes) and 26.42 (remaining genes) (Welch’s Two sample t-test t = 7.25, df = 244.84, p5.53e-12). Neighborhood connectivity is the size of the neighborhood that a focal node is in [47, 51]. Therefore in the PPI network analyzed here, we found the significant DE genes were from smaller neighborhoods, suggesting they have fewer interacting genes (and therefore edges). This conjecture was confirmed by the significantly smaller *degree* (number of edges) in ΔmtDNA ‘haplotype’ DE genes (top 200 ranked by p-value, Welch’s Two sample t-test: Females t = 3.5654, df = 246.75, *p* = 0.0004; Males t = 10.302, df = 409.05, *p* < 2.2e-16) compared to non-significant genes.

The ΔmtDNA ‘species’ sensitive genes and GxG- sensitive genes demonstrated qualitatively the same effects, and both neighborhood connectivity and degree measures were significantly smaller in the significant genes lists compared to the non-significant genes in both sexes.

The GGI and PPI network analyses present a consistent result that ΔmtDNA ‘haplotype’ sensitive genes are found in poor connectivity regions of networks, with relatively small numbers of edges. In a similar manner to the SAFE algorithm we devised a test to quantify the amount of mtDNA sensitivity in neighborhoods of genes. For this test, the nodes that connect directly to a focal gene are scored as their log (likelihood ratio) from the formal ΔmtDNA ‘haplotype’ analysis and the mean of all interactions was used to rank the focal genes. For this analysis we used a more comprehensive PPI network that was obtained from the Drosophila Interactions Database (DroID) [52]. A full description of the analysis appears in the Methods. The gene rankings for females and males can be found in Additional file 3: Table S3. The most highly ranked genes correspond with regions of the network that are inherently connected to mtDNA-sensitive genes and are not necessarily the DE genes themselves. This approach is likely to be more robust than a straightforward DE analysis because it uses prior systems information about known sub-networks to identify hotspots of mtDNA-sensitive genes. In the same way that we found genes that were physically co-expressed in clusters along the chromosomes, we now had the opportunity to test whether the interaction network is clustered with respect to mtDNA-sensitive genes.

**Fig. 5:**
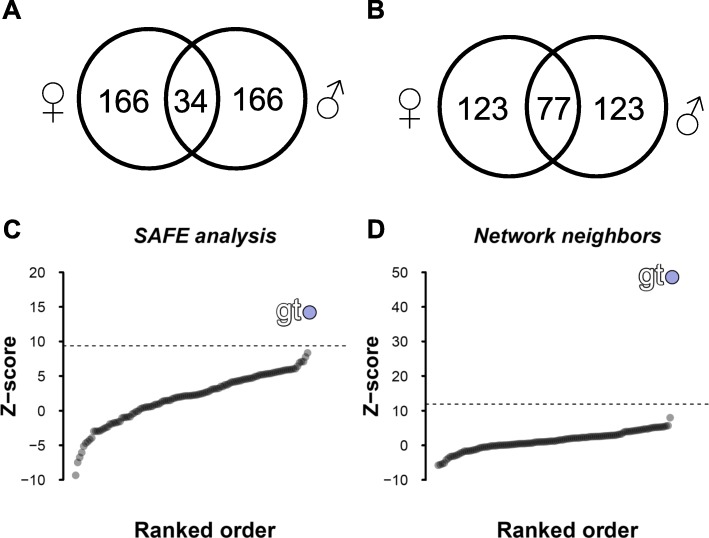
Female-male enriched gene overlap and transcription factor binding site enrichments. The 200 top ranked genes in both males and females were intersected for the SAFE (**a**) and neighborhood enrichment analyses (**b**). The overlapping proteins were analyzed for transcription factor binding site enrichment using oPOSSUM-v3 [53]. The SAFE analysis overlap (34 genes (**a** and **c**)) and the network neighborhood score overlap (77 genes (**b** and **d**)) were both highly enriched for the *giant* (*gt*) TFBS motif. Z-scores represent over-representation of the TFBS motif relative to a background set. The dashed horizontal lines in **d** and **e** represent the mean z-score + 2*SD

A gene ontology (GO) analysis of the 200 top-ranked focal genes in female neighborhoods revealed significant enrichment of the ‘electron transport chain’ GO process term (FDR q-value: 6.2e-45) and the ‘NADH dehydrogenase activity’ functional GO term (FDR q-value: 2.74e-26). In males, the same two terms -‘electron transport chain’ GO process (FDR q-value: 1.61e-41) and ‘NADH dehydrogenase activity’ GO function (FDR q-value: 2.28e-24) - were top-ranked. Clearly, top-ranked focal genes in both sexes are enriched for similar GO terms and between the genes lists there was considerable overlap between the sexes for the top 200 gene identities (intersection = 77 genes; Fig. 5b). It is noteworthy, however, that the GO categories were similar between the sexes, yet approximately two-thirds of the genes in the lists were unique to each sex, suggesting sex-specific gene neighborhood enrichments. The top two focal genes in both sexes were *CG4942* (a membrane insertase associated with the *Cox18* family [54]), and mitochondrial *Leucyl-tRNA synthetase*. Interestingly, a second membrane insertase (*CG6404*) was also the top 20 focal genes in both sexes.

We next conducted a transcription factor motif analysis to determine if the 77 shared genes between females and males were enriched for any binding site motifs that may indicate enrichment for regulatory sequences. Figure 5d shows the results of this analysis and reveals a strong signature for the basic leucine zipper factor *giant* (*gt*). Two of the 77 genes in the intersection (*bellweather* (*blw*) and *ATPsynthase gamma* (*ATPsynγ*)) are known to directly interact with giant. *Bellweather* encodes an alpha subunit of the mitochondrial F1F0 ATP synthase complex (complex V), the final enzyme of the oxidative phosphorylation pathway. *ATP synthase gamma* encodes an additional subunit of Complex V*.* We repeated this motif enrichment analysis on the intersected genes from the top 200 genes in the SAFE analysis (Fig. 5c) for which there was a 34 gene overlap across the sexes (Fig. 5a). Again, we identified *giant* as the highest ranked gene by z-score. Three of the 34 genes in the female-male intersection are known to interact with giant: *ATPsynthaseC* (*ATPsynC*), *blw* (see above), and *ATPsynγ* (see above).

The striking enrichment for the *giant* transcription factor binding site (TFBS) motif at high ranking ΔmtDNA ‘haplotype’ neighborhoods provides an opportunity to make some predictions about where mtDNA substitutions may affect transcript expression in the context of overall genome architecture. The *giant* gene is not differentially expressed across mtDNA haplotypes in either females (*p*-value = 0.524; FDR = 1.0) or males (*p*-value = 0.175; FDR = 1.0). To test the repeatability of the *giant* TFBS enrichment across alternative nuclear backgrounds, we performed SAFE enrichments using the same PPI network along with three different nuclear backgrounds from previous mitonuclear transcription investigations (*DGRP-820*, and *OregonR* and *AustriaW132*) [21, 30]. Across all four nuclear backgrounds, *giant* was the top ranking TFBS motif overall and was the number one rank in 4/8 background x sex combinations (Additional file 10: Figure S4). This result was qualitatively identical when median rank or mean rank was used, suggesting the *giant* association is robust across unrelated isogenic nuclear backgrounds. Since genome architecture is partly determined by co-expressed clusters of genes that have developmental stage-specific expression patterns in general, we next wanted to test whether the timing of a gene’s expression was associated with its clustering.

### Timing of gene expression during development is poorly correlated with the norms of reaction of mtDNA-sensitive genes

**Fig. 6:**
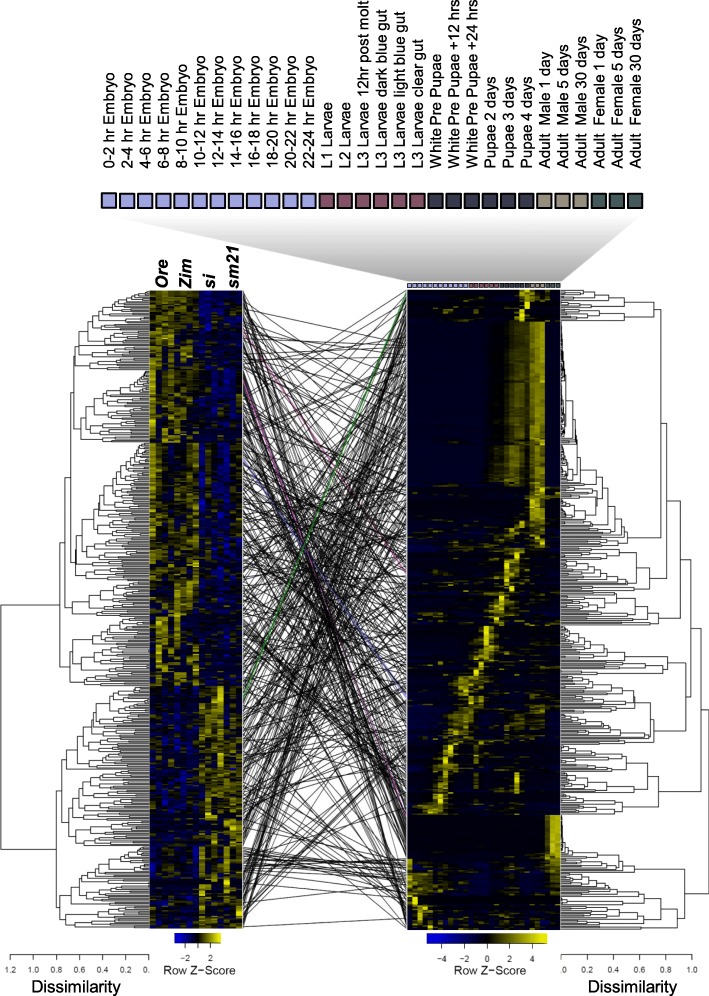
Tanglegram and heatmaps of the top 500 ΔmtDNA ‘species’ DE genes in females. Hierarchical clustering dendrograms and heatmaps of RNA-seq reaction norms (left) and development time expression (right) [55] are compared, revealing extensive crossing over and also consistent regions where there is phenetic resemblance between dendrograms (where parallel lines join genes with the same ID). DE genes are not limited to any particular ontogenetic gene expression period. Arbitrarily colored lines represent genes that are adjacent in both dendrograms. The same effects were evident when the top 50, 100, or 200 genes were used in the analysis. The order of genotypes in the leftmost heatmap (RNA-seq data) is *OreR*, *Zim53*, *si*I and *sm21* (left to right). The developmental time course is color coded: embryo (violet), larval (red), pupal (black), and male (sand) and females (green) adult stages are shown

We wanted to determine if there was correspondence, or more generally, overlap between the timing of a gene’s expression during development, and whether those same genes were sensitive to mtDNA variation as a 5 days old adult fly. In other words, we asked whether genes that are expressed at a particular developmental stage are more sensitive to mtDNA variation. We obtained *D. melanogaster* development stage-specific expression data from [55] and compared the dendrograms of gene expression patterns after hierarchical clustering, with clustering patterns obtained in our mtDNA-specific RNA-seq dataset. Figure 6 shows an example of the positioning of the top 500 genes ranked by *p*-value for the ΔmtDNA ‘species’ effect in females, with the corresponding genes in the development stage data set. To quantify the concordance between the sorting on dendrograms – the “phenetic resemblance” [56] - as a test of consistency, we estimated entanglement using the [dendextend] R package [57]. In this context, entanglement is a measure of the quality of the alignment of the two dendrograms and therefore is a proxy of the amount of crossing over between the alternative dendrograms. High entanglement scores (on a 0- > 1 scale) indicate poor similarity between dendrograms, while low entanglement indicates high concordance.

In all nine analysis comparisons, the top 500 genes ranked by *p*-value, were found in a mixture of development-stages and were not specific to embryo, larval, pupal, or adult stages. The example shown in Fig. 6 (entanglement = 0.75, post-*step2side* untangling = 0.22, see Methods for details) describes the female top 500 ΔmtDNA ‘species’ effect genes. The same qualitative effect is observed when the top 50, 100, or 200 genes are used (data not shown). The tanglegram (the lines connecting genes with the same ID) indicates there is some signal of similar sorting of the behavior of mtDNA-sensitive genes and their behavior during development. Groups of genes that are clustered in one dendrogram were also partially clustered in the other dendrogram and these are identified by more-or-less parallel lines in the tanglegram. This suggests that the norms of reaction of genes are to some degree associated with the behavior of that same gene over developmental time and therefore, haplotype-specific changes are being revealed at specific developmental stages associated with those genes. In this example the overall entanglement value was high (0.75) indicating a poor statistical support of concordance, even though there are clear clusters of genes showing resemblance between their reaction norm and the development stage of expression.

We calculated a second measure of similarity between the topologies of two hierarchical dendrograms using Goodman and Kruskal’s (G-K) γ [58], a value ranging from − 1 to + 1. Gamma is the probability of a consistent ranking minus the probability of an inconsistent ranking [59]. Values near zero indicate dendrograms that are not statistically similar in topology and γ = 1 is a perfect correlation. Following 100 permutations of γ against a null model of the same dendrogram but with shuffled leaves (gene IDs) we found zero support for ‘similarity’ between the dendrograms (γ = − 0.00574, 95% CI: − 0.048 to 0.043). The permutated γ values were significantly different from a perfect alignment (with γ =1, one-sided t-test: *p*-value~ 0) and significantly different from a random shuffling of dendrogram leaves (γ = − 0.3733, one-sided t-test: *p*-value~ 0). So, while the dendrograms are themselves dissimilar in topology, there is moderate entanglement between them, highlighting a potential for stage-specific expression that is altered by mtDNA variation. The cophenetic correlation was 0.307.

In the equivalent analysis in males, the entanglement value was 0.60 (0.23 after *step2side* optimization). The permutation test of the G-K gamma statistic revealed significant dissimilarity between dendrogram topologies (one-sided t-test against γ =1; *p*-value~ 0; one sided t-test against γ = 0.12; p-value~ 0; overall γ mean = − 0.0009, 95% CI: − 0.014 to 0.019); qualitatively the same result as in females. The cophenetic correlation was 0.05.

So far, the top genes in the ΔmtDNA ‘species’ effect list show some localized, but not general signal of correspondence with the developmental stage expression profile. Using the complete transcriptome data set [55], we next tested whether there was a signal of mtDNA-sensitivity after transcriptome-wide hierarchical clustering.

### Signal of mtDNA effects are clustered in different developmental stages across all transcripts

**Fig. 7:**
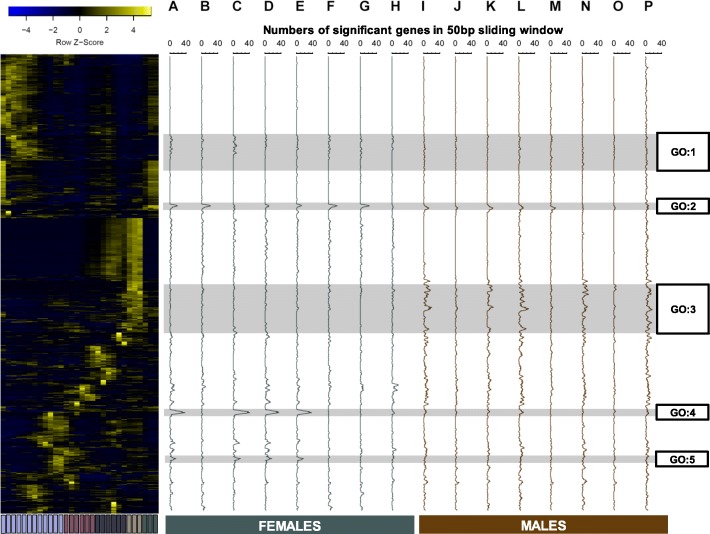
Haplotype contrasts show signals of temporal expression clustering that differ between contrast types and between the sexes. The heatmap represents a hierarchical clustering of the transcriptome (*n* = 12,007 genes) that could be compared between the current study and that of Graveley et al. (2010). Sliding window analyses (**A** to **P**) represent the numbers of genes that are found in the top 500 DE gene lists, ranked by p-value. The rolling sum was collected over a window size of 50 genes. Female (green: **A** to **H**) and male (brown: **I** to **P**) analyses are shown. Contrasts are reported as follows: ΔmtDNA ‘haplotype’ (**A**, **I**), ΔmtDNA ‘species’ (**B**, **J**), *Zim53-OreR* (**C, K**), *Zim53-si*I (**D, L**), *Zim53-sm21* (**E, M**), *OreR-si*I (**F, N**), *OreR-sm21* (**G, O**), and *si*I*-sm21* (**H, P**). Regions of interest are highlighted by GO: 1 – GO: 5 labels. Gene ontology analyses corresponding to these regions can be found in the Additional file 4: Table S4

We next performed a sliding window analysis across the whole transcriptome that had been sorted by hierarchical clustering. In this way, genes with mtDNA-sensitivity were mapped to the dendrogram of development stage expression. Using a sliding window of 50 ordered genes, we scored the rolling sum across the whole dendrogram. We identified several regions of the dendrogram with a strong signal of mtDNA-sensitivity enrichment (Fig. 7). Importantly, these regions are also associated with specific mtDNA pairwise contrasts, suggesting the unique polymorphisms between mtDNA haplotypes are not just changing the numbers of differentially expressed genes, but also identities of temporal co-expression hubs, consistent with the enriched regions of the GGI and PPI networks and the tanglegrams (see above). The genes in the enriched peaks were more likely than not to be found in the improper module of low-connectivity genes. Gene ontology analyses were conducted on a 400 gene window centered on the highest-peak gene in regions GO1-GO5 (Fig. 7; Additional file 5: Table S5). Figure 7A-P describes the mtDNA contrasts, and there are clear sex- and haplotype-contrast-specific cluster peaks. Distinct haplotype contrasts (and therefore the variants the contrasts expose) are clearly associated with different windows of gene expression in the developmental time course.

### Essential (housekeeping) genes are underrepresented in mtDNA-sensitive gene sets

We have shown that nuclear genes whose expression is altered by mtDNA background tend not to be hubs in PPI and GGI and are enriched in the set of genes that show low co-expression. We finally tested whether known housekeeping genes were associated with variation in mtDNA. We used two datasets to test this hypothesis: Three Drosophila housekeeping gene cluster types identified by Weber and Hurst [60] and the Online GEne Essentiality database (OGEE: http://ogee.medgenius.info/browse/).

The top 500 ΔmtDNA ‘haplotype’-sensitive genes were statistically underrepresented in the ‘breadth’ clusters [60], containing genes that are expressed in all 14 adult tissues: females (6.1% of expected, Hypergeometric test: *p* = 5.00e-27) and males (20.6% of expected, Hypergeometric test: *p* = 2.53e-16). The Tau clusters, which also contain low-specificity housekeeping genes, were also statistically underrepresented in females (5% of expected, Hypergeometric test: *p* = 1.06e-14) and males (7.6% of expected, Hypergeometric test: *p* = 1.49e-13). A third cluster type, defined by genes with high levels of co-expression and functional coordination, showed evidence of statistical underrepresentation in females (60.3% of expected, Hypergeometric test: *p* = 0.0028), but no underrepresentation in males (78.3% of expected, Hypergeometric test: *p* = 0.22).

ΔmtDNA ‘species’-sensitive genes demonstrated qualitatively the same under-enrichments except for the small (third) cluster in females, which showed no statistical difference from the expected (89.9% of expected, *p* = 0.67). The broad-scale mitonuclear (GxG) genes were underrepresented in the breadth clusters (females: 53.3% of expected, Hypergeometric test: *p* = 0.0001; males: 41.1% of expected Hypergeometric test: *p* = 1.14e-07) and the tau clusters (females: 22.7% of expected, Hypergeometric test: *p* = 1.33e-08; males: 30.2% of expected Hypergeometric test: *p* = 7.21e-07), but showed no statistical under- or over-enrichment in the small clusters (females: 97.6% of expected, Hypergeometric test: *p* = 0.89; males: 78.3% of expected Hypergeometric test: *p* = 0.22). These results are largely supported by other mitonuclear genotypes from previous studies [21, 30], which show under-enrichment of housekeeping genes in mtDNA-sensitive gene lists across multiple nuclear backgrounds and in both sexes (Additional file 5: Table S5).

In the second essential gene data set, there were two cases of significant departure from the expected numbers of genes given the gene list sizes. The length of the essential genes list that could also be found in the DE gene lists was 294. In the intersection of the top 500 genes ranked by *p*-value, the female ΔmtDNA ‘species’ list was significantly different from the expected number (48.1% of expected, Hypergeometric test: *p* = 0.04), and the male ΔmtDNA ‘haplotype’ was significantly underrepresented (16.0% of expected, Hypergeometric test: *p* < 0.001). All remaining intersections were not significantly different from expected (Hypergeometric test: *P* > 0.05 in all cases).

## Discussion

The interaction between mtDNA- and nuclear-encoded genes and their products is a mainstay of eukaryotic life, and understanding how mtDNA variation can influence the behavior of the interactome is a fundamental goal in quantitative genetics. Here, we show that mtDNA variation alters nuclear gene expression unequivocally in both sexes, has a strong signature of gene co-regulation components and gene clustering, and is modified by broadscale mitonuclear epistasis. Altogether, we show that there are predictive patterns to mtDNA-sensitivity and core regions (and individual genes) of the interactome that are sensitive to mtDNA variation. More importantly, we also show for the first time in any species that the most sensitive genes to mtDNA variation are found in poorly connected regions of GGI and PPI networks, with non-housekeeping roles. Examination of mtDNA-sensitive genes lists reveals an abundance of ETC complex genes, and this association with OXPHOS is perhaps not surprising since we have deliberately disrupted a co-evolved gene-gene and protein-protein interacting complex. That we recover OXPHOS-related genes, and that there is an underlying signature of transcription factor binding motifs, we can suggest that *giant* associated sequences may be predictive sites for ΔmtDNA effects.

In both sexes we also found common genes among the mtDNA-sensitive network neighborhoods. Mitochondrial Cox18 membrane insertases (*CG4942*(top-rank) and *CG6404*) were common in the top 20 neighborhoods in both males and females, and have a major role in the assembly of respiratory chain complex IV [61]. The second ranked hub gene in both sexes was a mitochondrial Leucyl-tRNA synthetase (*LeuRS-m*). tRNA synthetases are involved in mitochondrial translation and the ligation of amino acids to their cognate tRNAs in mitochondria. We have previously identified nuclear-encoded *tyrosyl-tRNA synthetase* as a causal nuclear gene that interacts epistatically with polymorphism in its respective mitochondrial *tRNA*^*Tyr*^ in Drosophila [19]. The negative epistasis phenocopies a mitochondrial translation defect and causes a range of deleterious traits, effectively mimicking a mitochondrial disease.

In *D. melanogaster* and *D. simulans*, the mtDNA *tRNA Leu*^*(CUN)*^ is flanked by the *long ribosomal RNA* and *ND1* genes, and overlaps a known rRNA transcription terminator sequence [62]. Between Drosophila species [62] and the haplotypes used in this study [63], the rRNA transcription termination sequence is fully conserved. Transcription of the neighboring ribosomal genes (found on the same polycistronic transcript as *tRNA Leu*^*(CUN)*^) is estimated to be up to seven times higher than genes clustered downstream of the transcription termination site [64]. It follows that the co-transcription of *tRNA Leu* ^*(CUN)*^ with neighboring ribosomal genes may allow its abundance to act as a proxy of ribosomal RNA levels, and provide a small molecule signaling component to gene regulation stoichiometry.

In humans, *Leucyl-tRNA synthetase* is a key mediator for amino acid signaling to mammalian target of rapamycin (mTOR) [65]; a major protein kinase involved in protein translation, regulation of cell size, autophagy, and a mediator of energy balance via transcriptional control of mitochondrial function [66]. It is perhaps unsurprising that genetic interactors of mTOR are themselves sensitive to mtDNA mutations, since mitochondria are deeply associated with protein translation and considerably overlap with the functions of mTOR. Our results suggest that mtDNA mutations are likely to indirectly (sensu ‘omnigenic’ model [67]) or directly affect systems-level functions of mitochondria via mTOR and its interactors, a phenomenon we have previously reported in a mtDNA introgression model [68]. In that study it was noted that the benefits of rapamycin to the cell are highly dependent on the mtDNA genotype, once again reinforcing the genetic context-specificity of rapamycin on mitochondrial function.

The association between the interactors of *Leucyl-tRNA synthetase* and highly differentially expressed genes in the present study suggests tRNA synthetases may mediate a common syndrome of mtDNA variation. That is, tRNA synthetases and their interactors are highly sensitive to polymorphism in their cognate tRNAs. This mechanism requires mtDNA polymorphism in the respective tRNA to disrupt efficient communication with the synthetase protein, for example during aminoacylation [69]. So, are there polymorphisms in one or both of the mt *tRNA*^*Leu*^ genes between haplotypes in this study system? Yes- one of the mtDNA *tRNA*^*Leu*^ molecules (*tRNA*^*Leu(UUR)*^~ nt 3014–3079) is monomorphic, but the other (*tRNA*^*Leu(CUN)*^~ nt 12,697–12,761) contains two polymorphisms: a species-specific SNP that delineates *OregonR* (Genbank accession number AF200828.1) and *Zimbabwe53* (AF200829.1) from the *D. simulans* haplotypes; and a second SNP that delineates *si*I (AF200834.1) from *sm21* (AF200841.1) (data not shown, alignments from [63]). The first polymorphism occurs in the variable loop and the second occurs in the TΨ loop. Altogether, there is clear potential for *tRNA*^*Leu(CUN)*^ mutations to be associated with systems-wide gene expression, although this may not necessarily require mitonuclear interaction and could be a phenomenon of mtDNA variation per se. Future work should investigate this possible link.

Transfer RNAs are among the most conserved sequences on the mtDNA molecule, yet they are hotspots for pathological mutations [70, 71] with approximately two-thirds of human pathological mtDNA mutations occurring in one tenth of the mitochondrial genome; occupied by tRNAs [72, 73]. One of the first human diseases linked to a tRNA mutation was identified in the mitochondrial *tRNA*^*Leu(UUR)*^ gene, and is associated with mitochondrial encephalomyopathy, lactic acidosis, and stroke-like episodes (MELAS) [74]. Furthermore, mutations in *tRNA*^*Leu(CUN)*^ have been associated with skeletal and eye muscle disorders [75, 76], isolated skeletal myopathy [77], exercise intolerance [78], and cardiomyopathy in humans [79]. The inherent coupling of mtDNA tRNAs and their respective synthetase proteins from the nuclear genome provides a mitonuclear epistasis model that can be used to dissect the role of this dual-encoded PPI system.

We have previously shown that there is appreciable developmental time variation across the genotypes used in this study, with *si*I*;DGRP-315* demonstrating the slowest development time [20]. Future work should aim to determine if the underlying genetic patterns of co-variation that are revealed at the systems biology level are useful predictors of fine-scale epistatic interactions that influence whole organism phenotypes.

### Non-coding RNAs and clusters of mtDNA-sensitive genes

We found that nuclear genes that are sensitive to mtDNA variation are overrepresented as physical contiguous clusters on chromosome arms. Interestingly, these clusters were often flanked by non-coding and antisense RNAs, suggesting a possible mtDNA-related regulatory role of these non-coding genes. Protein coding genes constitute only a small fraction of transcribed DNA (1–2% in humans: [80]) (20% in *D. melanogaster* [55]) and non-coding RNAs are important factors of gene regulation and epigenetics in mammals [81] and Drosophila [55]. Specifically, large intergenic non-coding RNAs play an important role in guiding chromatin-modifying complexes to specific loci [82], and can be modified by environmental factors (e.g. low protein diet) that can also be sex-specific [83, 84].

Here, we propose that the physical proximity of non-coding RNAs to mtDNA-sensitive clusters is good evidence that these untranslated sequences may be important genetic landmarks for trans-acting factors associated with mtDNA variation. In males, we found a cluster of *Turandot* genes on 3R were significantly altered by ΔmtDNA. *Turandot* (*Tot*) genes are a family of eight stress induced humoral factors that are found in three locations around the *D. melanogaster* genome [85]. *TotA* encodes a peptide that is secreted into the haemolymph from the fat body and makes the organism more resistant to a myriad of stresses, including bacterial challenge, high temperature, mechanical pressure, dehydration, UV irradiation, and oxidative agents [86]. It is likely that all eight proteins in the *Turandot* family respond to stress in the fat body via the JAK-STAT pathway [87]. Not only is there a significant cluster of ΔmtDNA sensitive genes on the 3R chromosome (*TotA*, *TotC*, *TotX*), but a fourth *TotM* gene is also significant and found on the 2 L chromosome arm. This highly coordinated gene family response to mtDNA variation may imply that mitochondrial variation is associated with bacterial response pathways.

In some cases, genes that were highly ranked neighborhood hubs were fully nested in long, non-coding RNAs (e.g. *CG33229*, the third ranked hub gene, fully nested in *lncRNA: CR42862*). We do not have the resolution at this stage to describe the importance of non-coding RNAs, but we show strong evidence that non-random associations of mtDNA-sensitive clustered genes are physically linked to non-coding RNA.

In both co-expression gene clustering (GGI) and neighborhood connectivity (PPI) analyses, low connectivity and low edge numbers were associated with mtDNA-sensitive genes. This is perhaps not surprising because theoretical derivations of WGCNA show proper modules have high average clustering coefficients when compared with improper modules [47], and proper modules sparsely overlap with mtDNA-sensitive genes. Therefore the mtDNA-sensitive genes are, by definition, more likely to have relatively low-connectivity and relatively low edge numbers. Nevertheless, our GGI and PPI analyses independently provide good support that mtDNA-sensitive genes are enriched in low connectivity regions of GGI and PPI networks. The PPI network [49] we used as the topology of our network parameter analysis (clustering coefficients and degree) is likely to be collinear with a large amount of the GGI network we established. Since both analyses consistently describe the same effect, we should consider this systems biological approach to mtDNA genetic effects as a powerful tool to dissect the regional enrichments of mtDNA-sensitive genes.

High connectivity (hub) genes generally show low expression variance and are under higher constraint than low connectivity genes [88]. Likewise, housekeeping genes that show low tissue specificity have typically high clustering coefficients [89] . Taken together, these observations are consistent with our finding of low-connectivity gene enrichments in our significant mtDNA-sensitive gene lists, and an underrepresentation of housekeeping genes.

### Specific haplotype contrasts show specific transcriptome responses

One of the illuminating findings from this study is that pairwise contrasts between haplotypes demonstrate both quantitative and qualitative differences in their abundance and network positioning. In other words, the polymorphisms that delineate haplotypes show a propensity to affect different gene sets that have different temporal patterns of expression and are found in different regions of PPIs. The patterns of gene expression that we observed in various contexts are not necessarily independent. For example, genes that are physically interacting are more likely than not to be found nearby in a hierarchical clustering of temporal expression pattern or quite possibly, physically clustered on chromosomes [45]. The main purpose of this study was to define *predictive* properties of mtDNA-sensitive genes, and a much clearer picture emerges when several pieces of evidence are compiled.

We found robust evidence that the highest ranking gene hubs associated with mtDNA effects across the sexes share common transcription factor binding sites and there was a strong signature of enrichment from the *giant* (*gt*) transcription factor. Interestingly, the position weight matrix of the *giant* binding site motif is ATTACGTAAT [90]; a hairpin compatible sequence that has the potential to form cruciform structures [91], which themselves have been implicated in gene expression regulation. The same *cis*-regulatory motif has previously been identified in Drosophila nuclear OXPHOS genes [92] and highlights that genes that are surrounded by mtDNA-sensitive genes are likely to also be nuclear OXPHOS genes themselves.

### Non-random genomic associations and mtDNA-sensitive genes

We performed several tests to determine if mtDNA-sensitive genes are non-random with respect to their physical positioning on chromosomes, their temporal expression during development, and their physical location in GGI and PPI networks. In all tests, we found good evidence that mtDNA-sensitive genes are not randomly distributed in any of the above scenarios. In fact, the representation of tandem duplicates, and similar norms of reaction for closely physically linked genes suggests there are central regions of co-regulation. In mtDNA genes, however, the rules are different. The similar norms of reaction of adjacent genes can be explained by the generation of polycistronic mRNAs during transcription [93], since the mitochondrion is transcriptionally a prokaryote with large polycistronic transcripts. Alternatively, RNA-seq read mapping biases generated by mapping mtDNA sequences to a known, sometimes highly dissimilar sequence, may contribute to the ‘species’ effects in mtDNA genes. In this study we have therefore focused our interpretation on the nuclear genes that are presumably under some retrograde signaling from the mtDNA or mitochondrion.

## Conclusions

It is challenging to tease apart the major associations of mtDNA sensitive genes in this study partly because of their collinear nature. Expression timing, spatial expression, housekeeping roles, and physical location etc. are all inextricably linked because the genome’s architecture has been shaped over evolutionary time by necessity of spatial-temporal expression patterns [60]. What we do show, however, is that core regions of the interactome are sensitive to ΔmtDNA, and these gene sets are often consistent across the sexes, with underlying co-regulatory signatures. This is a major development for predicting the most likely sources of mitonuclear incompatibilities in, for example, patients undergoing mitochondrial replacement therapy. We show here that genes disrupted by ΔmtDNA are most likely to be found in the improper module, have transcription factor binding site enrichments for the *giant* TF, are statistically enriched in clusters, underrepresented in housekeeping genes, and are often associated with non-coding RNAs. Future work should aim to further dissect the identity of trans-acting factors that mediate the cross-talk between nDNA and mtDNA genomes, and determine whether the results we have found at the whole organism level are found in individual tissues. The possibility of mosaic effects across different tissue types would suggest our results here may be conservative. It remains to be seen how individual cells and tissues respond to ΔmtDNA. A more comprehensive understanding at the cell and tissue levels is necessary to improve the accuracy of anticipated effects of mtDNA mutation.

## Methods

### Fruit fly genotypes and husbandry

The strains used in the current study are a sub-set of a larger panel of mitonuclear genotypes constructed using the Drosophila Genetic Reference Panel (DGRP) and six phylogenetically distinct mtDNA haplotypes [20]. DGRP strains were obtained from the Bloomington Drosophila Stock Center, Indiana University. Flies were generated by mitonuclear introgression using precise balancer chromosome extraction (see Zhu et al. 2014 for crossing scheme; Mossman et al. 2016 for details). Male flies from the original DGRP stocks were then backcrossed to virgin females of the newly constructed mitonuclear strains for greater than five generations to eliminate residual nuclear heterozygosity that may have been maintained during the chromosome substitutions. In the current study we selected the *DGRP-315* (RRID:BDSC_25181) and *DGRP-820* (RRID:BDSC_25208) nuclear backgrounds along with two *D. melanogaster* mtDNA haplotypes: (i) *Zimbabwe53*, (ii) *OregonR*; and two *D. simulans* haplotypes: (iii) *si*I, and (iv) *sm21*. We selected these genotypes based on their development time phenotypic scores in the previous study. The selected genotypes therefore do not represent a random sample of mitonuclear variation in Drosophila. However, as we wanted to characterize haplotype and G x G effects on gene expression we theorized that such effects would be more likely to be detected in genotypes with known whole organism phenotypic variation. For the G x G interactions, we focused on a 2 mtDNA × 2 nDNA experimental design as we have found this to be sufficient to capture genes whose expression is sensitive to mitonuclear effects [21]. The G x G experimental haplotypes were *Zimbabwe53* from *D. melanogaster*, and *sm21* from *D. simulans* on *DGRP-315* and *DGRP-820* nuclear backgrounds. Our standard reporting of mito;nuclear genotypes is the mtDNA haplotype followed by the nuclear background (e.g. *Zim53;DGRP-315* is the *Zimbabwe53* mtDNA haplotype on the *DGRP-315* nuclear background). The mtDNA sequence divergence estimates are reported in a previous publication [18]. Briefly, there are up to 103 amino acid substitutions between the mtDNA contrasts in the current study, and up to 438 synonymous substitutions. The numbers of fixed differences between species mtDNAs are much lower in magnitude.

Flies of each mitonuclear genotype were reared under standard laboratory conditions in a controlled environment room on 12 h light: 12 h dark cycles at a constant 25 °C. Prior to the experimental setup, flies were maintained in density-controlled bottles for two generations to minimize condition-dependent carry-over effects on offspring traits. When the experimental cohort was ready to be studied, newly eclosed virgin males and females were collected together and held in bottles with a cornmeal-yeast food (quantities per 200 ml food - Agar: 1 g, SAF yeast: 20 g, Yellow cornmeal: 9 g, Sucrose: 20 g, Tegosept: 0.45 g dissolved in 95% ethanol 4.5 ml, distilled H_2_O to 200 ml total volume) for 3 days. After this holding time, males and females were separated by sex and held in same-sex vials of the same food type for 2 days until they were flash frozen in liquid nitrogen and stored at − 80 °C prior to RNA extraction. The flies used for RNA sequencing were 5 day old adults at time of RNA extraction.

### Wolbachia elimination

One hundred and eight out of the 205 DGRP genotypes are known to contain *Wolbachia pipientis* [94], a bacterial endosymbiont that is maternally transmitted and that can confer altered phenotypes. The infection status of the original *DGRP-315* strain is negative, and the *DGRP-820* is positive. To eliminate any cofounding effects of *Wolbachia* in our flies, larvae (and subsequent adults) were cultured on Instant Carolina Media with 0.03% tetracycline for two generations. Strains were then screened for *Wolbachia* infection status using two *Wolbachia*-specific primer pairs: (i) 1F, 5′- ttgtagcctgctatggataact-3′, 1R, 5′- gaataggtatgattttcatgt-3′ and (ii) 2F, 5′-tgtggtgccagagtacttgaa-3′, 2R, 5′-gctttataagcgcgttcagc-3′. *Wolbachia*-positive controls were run in the same PCRs and failure of samples to amplify either PCR product was evidence of *Wolbachia*-negative status. All strains were confirmed as *Wolbachia-*negative prior to this study.

### RNA extraction

Total RNA was extracted from batches of 30 whole flies per biological replicate (× 4) per strain in both sexes. Each biological replicate was sourced from an independent rearing bottle. Whole flies were initially homogenized using a Qiagen TissueLyzer (Qiagen). Total RNA was extracted using Qiagen RNeasy mini Kits (Qiagen) following manufacturer’s instructions. Total RNA extractions were stored at − 80 °C before submission for mRNA molecular preparation and sequencing with Genewiz (Genewiz, South Plainfield, NJ), using their in-house pipeline for 50 bp single-end reads on the Illumina Hiseq 2500 platform.

### Sequence read analysis

Sample preprocessing was performed using computational resources from the Brown University Center for Computation and Visualization (CCV). Fastq files were assessed for quality control measures using the FastQC program (fastqc/0.10.1) (https://www.bioinformatics.babraham.ac.uk/projects/fastqc/). Libraries were then filtered for low quality reads using the FASTX toolkit (v2.6) (http://hannonlab.cshl.edu/fastx_toolkit/), specifically the fastq_quality_filter with -q 20 (minimum quality phred score 20) and -p 80 (minimum percentage of bases that must have the –q score). For example, 80% of reads in a sequence must have at least a minimum phred score of 20 to pass quality filtering. Truseq adapters were then clipped from the sequences using the fastx_clipper program implemented in the FASTX toolkit (v2.6) (http://hannonlab.cshl.edu/fastx_toolkit/commandline.html). Sequence reads were then mapped to the Drosophila dm3 reference genome using TopHat (v2.0.12) [95] and Bowtie2 (v2.2.3) [96] using the flags -p 16 -i 30 -I 20000 --segment-length 25 and the dm3flybase.gtf annotation file obtained from the University of California Santa Cruz Browser (https://genome.ucsc.edu/) [97]. BAM files were converted to. SAM files using samtools (v0.1.19) [98] and sequences were counted at annotated genome features using htseq-count implemented in the HTSeq program [99]. Read counts at gene features were used for downstream analyses of mRNA expression.

### RNA-seq data analysis

We used the *edge*R package [40] on the read count data (see above) to formally detect significantly differentially expressed (DE) genes. Multiple test correction was performed using the Benjamini-Hochberg method [42] and a False Discovery Rate (FDR < 0.05) was used unless otherwise stated. To test whether mtDNA haplotype per se was associated with gene expression, we performed Analysis of Deviance-type contrasts. To test for pairwise mtDNA haplotype contrasts within each sex, the full model (including all haplotypes) was fit using gene dispersion parameters based on estimateGLMCommonDisp, then estimateGLMTrendedDisp, then estimateGLMTagwiseDisp as described in the edgeR vignette (https://bioconductor.org/packages/release/bioc/vignettes/edgeR/inst/doc/edgeRUsersGuide.pdf). Contrasts for all pairwise comparisons were performed within each sex to test whether genetic distance between mtDNA molecules was associated with the numbers of DE genes. Contrasts were made to test for mtDNA ‘species’ effects and broadscale mtDNA x nDNA interactions (GxG). In the latter test, the (*Zim53-sm21*) contrast in the DGRP-315 background was contrasted against the (*Zim53-sm21*) contrast in the DGRP-820 background. This was repeated in both sexes.

### Manhattan plots of DE significance

To determine whether there was genomic location structure in ΔmtDNA haplotype, ΔmtDNA species, and GxG DE genes (expression quantitative trait loci: eQTLs), we downloaded the genomic coordinates of all genes in the analyses from Flybase using the batch download tool (http://flybase.org/batchdownload). Chromosome locations of genes were linearized with respect to their mean (middle nucleotide) gene coordinates. We plotted the –log_10_(*p*-value) of the respective DE analysis with the physical gene location.

### DGRP WGCNA analysis

To test if differences between mtDNA haplotypes (ΔmtDNA), or mtDNAs from different species (ΔmtDNA ‘species’), and mitonuclear (GxG) DE genes were associated with internal or external regions of gene-gene interaction (GGI) networks, we first mapped these genes to an independent empirical GGI network. Externally-sourced *D. melanogaster* gene expression profiles from 185 replicated DGRP genotypes in both sexes were obtained from the DGRP2 website (http://dgrp2.gnets.ncsu.edu/) and were clustered in an unsupervised manner using the Weighted Gene Co-expression Network Analysis (WGCNA) R package [100]. We used the independently obtained gene co-expression networks from the DGRP resource to map our significantly differentially expressed genes to circumvent the network topologies being influenced by our experimental design (see main text). WGCNA networks were constructed using the following user-supplied parameters: power = 15 (based on the soft threshold analysis ≥0.9), merging threshold = 0.0, network type = ‘unsigned’, max block size = 1000, minimum module size = 30). For the Cytoscape display figures (Additional file 7: Figure S1), gene interactions were restricted to those with a weight threshold > 0.05 (only well supported gene-gene interactions were plotted). Preceding the blockwiseModules command, we set a random seed (10913) for future replication.

### Protein-protein interaction (PPI) network

We obtained a signed, functional protein-protein interaction network (Table S15 in [49] to test for functional enrichment of our DE genes in a spatial context using the Spatial Analysis of Functional Enrichment (SAFE) package [48] and Cytoscape (v3.6.1) [101] plug-in. The PPI network was constructed in Cytoscape using the *prefuse force directed* layout algorithm and enriched regions of DE genes were calculated based on the SAFE enrichment score. The ‘attribute’ that was mapped to the network was the –log_10_(*P*-value) of a gene in an *edgeR* contrast of interest (see above).

Network analysis was performed on all genes in the Vinayagaman et al. (2013) PPI network to determine if any network parameters (average shortest path length, betweeness centrality, closeness centrality, clustering coefficient, eccentricity, neighborhood connectivity, radiality, stress, and the topological coefficient) were associated with DE in the three analysis classes (ΔmtDNA haplotype, ΔmtDNA species, and G x G). To conduct this we used the NetworkAnalyzer [51] network analysis tool implemented in Cytoscape with the ‘undirected network’ selection. We only report the results of the *neighborhood connectivity* and *degree* variables in the Results section due to redundancy of other (collinear) variables.

### Focal gene neighbor enrichment

To test whether network connectivity to neighboring genes is associated with any transcription factor binding site signatures, we downloaded and analyzed a PPI network from the Drosophila Interactions Database (DroID) [52] (version 2018_08, downloaded September 2018, dataset: ‘Gene Expression Correlation and Confidence Scores for physical protein-protein interactions’; http://www.droidb.org/data/DroID_v2018_08/confidence_correlation.txt).

This dataset includes the PPI from [49] but also includes additional PPI datasets from experimentally derived physical protein interactions from the databases: BioGRID (https://thebiogrid.org/), IntAct (https://www.ebi.ac.uk/intact/), MINT (https://mint.bio.uniroma2.it/) and BIND (http://bind.ca). These PPIs include additional interactions that increase the power to detect network neighbors that are differentially expressed. The main motivation of this analysis was to find hub proteins across a more comprehensive PPI network that are known to interact with proteins whose encoding genes are DE by ΔmtDNA. To perform this analysis the PPI network was first reciprocally inverted (e.g. an A- > B interaction is equal to a B- > A interaction). This way all focal genes would be assessed for the strength of the mtDNA DE effect of their known interactors. Interactions involving micro RNAs (miRNAs) were then removed, since these were not assayed in the RNA-seq analysis. In the final step, interactions without a confidence score, along with duplicate interactions were removed. The network used includes 210,486 edges and 8879 nodes (genes).

Differential expression of focal gene interactors was then assessed as the mean likelihood ratio (LR) of all known interactors based on the DroID PPI network (see above Fig. 5). Therefore for a given protein of gene A, that interacts with proteins of genes B, C and D, the mean LR values of genes B, C, and D were tabulated and ranked based on their mean LR value. High mean LR values are associated with genes that are directly linked to sub-networks of highly DE genes. The top 200 genes of these lists in females were intersected with the top 200 genes in males to find conservative genes that are hubs of DE across both sexes. This analysis was performed on the LRs of the ΔmtDNA haplotype, ΔmtDNA species, and G x G analyses.

All focal proteins (8879 in total) were assessed for their neighborhood ΔmtDNA effects. Proteins with low numbers of interactors could have a disproportionate influence on the mean value if their interactors had high (or low) LRs. While this effect is likely ‘biological’ we were cognizant that the number of interactors could influence the mean in a large way. To test this possibility, we conducted a sensitivity analysis on the minimum number of interactors a focal protein could have and this parameter had no qualitative effect on the top-ranked transcription factor highlighted by oPPOSSUM-3.0 [53] (data not shown). Furthermore, there was no relationship between the total number of interactors and the mean neighborhood LR (e.g. female mtDNA haplotype effect: *r* = 0.01, *df* = 8850, *P* > 0.05). We were therefore confident that our TF motif analysis was conservative and showed little sensitivity to the number of interactors considered.

### Housekeeping gene clusters, essential genes, and ΔmtDNA

We obtained our gene expression measures based on whole fly RNAseq and therefore needed to cross reference with tissue-specific datasets to align our results with known housekeeping genes that show expression across tissue types in Drosophila. To determine whether ‘housekeeping’ genes and ‘essential’ genes were enriched, indifferent, or underrepresented in the mtDNA contrasts, we downloaded known clusters of genes [60] that are physically localized in *D. melanogaster* and are consistent with housekeeping roles (demonstrate low tissue specificity or expression across all tissues), since their expression is abundant across many tissues. In Weber and Hurst’s (2011) analysis they identified three gene cluster types corresponding with: ‘Large’ clusters that contain functionally unrelated housekeeping genes, ‘Tau’ clusters that contain low tissue-specificity genes and ‘Small’ clusters that contain genes with high levels of co-expression that are functionally coordinated. We intersected the top 500 genes identified in the three DE analysis types in each sex with the known clusters in Weber and Hurst (2011). The results of the intersection correspond to the ‘realized’ intersections that we measured in our analyses. We also tested whether our realized intersections were different from a random genome-wide expectation. That is, for a random sample of 500 genes in the genome, how many housekeeping genes identified in [60] would we expect to intersect by chance? We permuted this 10,000 times with randomly sampled gene sets. To formally test for evidence of enrichment, we used a hypergeometric distribution test, *phyper*, implemented in the [stats] R package to calculate the probability of a realized overlap occurring by chance.

To test whether ‘essential’ genes are enriched or underrepresented in our DE gene sets, we downloaded an essential/non-essential gene list from the Online GEne Essentiality database (OGEE: http://ogee.medgenius.info/browse/) for *D. melanogaster*. We performed intersections between empirically-identified ‘essential’ genes with our realized DE gene lists. We focused on the genes that could be found in both DE and gene essentiality datasets (12,090 genes in total) [102, 103], identifying 294 ‘essential genes’ as a test set. Hypergeometric tests were used to formally test for over- or under-enrichment, as above.

### Developmental stage-specific enrichments

To test whether gene expression at particular developmental stages was associated with DE genes, e.g. are mtDNA-sensitive genes clustered in time in the fruit fly, we performed dendrogram comparisons of gene expression based on the current study, with developmental stage gene expression obtained from the literature [55]. We used the *dendextend* R package on clustered gene expression profiles to compare dendrograms for overlap and entanglement. Entanglement is an index between 0 and 1 and is a proxy for the amount of crossing over between dendrograms. High values represent highly entangled dendrograms, with low congruence, and vice versa. We report the results of the top 500 genes ranked by *p*-value for the entanglement analysis. The results for the top 50, 100 and 200 genes were qualitatively similar of the top 500 gene analyses (data not shown). We assessed congruence using: (i) unaltered dendrograms, and (ii) dendrograms constructed using a greedy forward step wise rotation approach to find a more optimal alignment solution (*step2side* entanglement). We further calculated Goodman and Kruskal’s gamma statistic [58, 59]; a measure of similarity between two hierarchical dendrograms, and the cophenetic correlation [56] with complete linkage to test for evidence of significant similarity (correlation) between gene orders across two focal dendrograms. The algorithms were implemented in the dendextend R package [57]. We performed a permutation test to calculate the statistical significance of the Goodman and Kruskal’s gamma index distribution against the null hypothesis of no similarity [57].

To look for non-random clusters of gene expression, we performed hierarchical clustering on the developmental time data across all available transcripts. Using this hierarchical clustered gene order we asked whether a dendrogram ‘leaf’ was a ‘top 500 significant’ gene across all comparison types. Intersected genes were scored as ‘1’, while non-intersected genes were scored as ‘0’. We then performed a sliding window analysis across the dendrogram to count the number of positive intersections with our significant gene list. The rolling sum of a 50 bp window was calculated using the *rollsum* function in the <zoo> R package. The same analysis was performed on a hierarchical clustering of tissue-specific gene expression to interrogate clusters of significant genes. Regions of interest were tested for Gene Ontology (GO) enrichments using the Gorilla Gene enrichment tool [104].

### Transcription factor binding site enrichment

oPOSSUM-3.0 was used to test for transcription factor binding motif overrepresentation near genes of interest identified in SAFE [48] and neighborhood connectivity analyses. The scanning parameters were: + 1000 bp/− 1000 bp of the transcription start site (TSS), and a minimum of 85% sequence similarity between the transcription factor binding motif and the Ensemble v64 dm3 annotated genome sequence, obtained from the UCSC Genome Browser [97]. All remaining parameters were kept as default and all 14,832 genes in the oPOSSUM3.0 database were used as the background gene set. Transcription factors were ranked by their ascending z-score and plotted to illuminate overrepresented TFBS. Binding site motifs were informed by the JASPAR transcription factor binding site profiles [90]. Position weight matrices from JASPAR were used to test for secondary DNA strand folding structures using Mfold [105] and a folding temperature of 25 °C (the temperature the flies were maintained at during this study). All remaining parameters were kept as default values.

### Statistical analyses

All statistical analyses and data visualizations were performed using R (v3.1.5) [106]. Library preprocessing was conducted on the Brown University CCV cluster.

## Additional files

Additional file 1: **Table S1.** Genes that are DE by ΔmtDNA ‘haplotype’ (FDR < 0.05). Genes that are private to each sex and shared between sexes are shown. Annotation symbols, Flybase IDs, cytogenetic map locations, chromosome arms, strand, gene symbols, names and the sex that the gene is significant in are shown. Genes that are shared between the sexes are marked as ‘both’ and are in gray boxes. FDR values are shown for females and males. (XLSX 24 kb)

Additional file 2: **Table S2.** Genes that are DE by ΔmtDNA ‘species’ (FDR < 0.05). Genes that are private to each sex and shared between sexes are shown. Annotation symbols, Flybase IDs, cytogenetic map locations, chromosome arms, strand, gene symbols, names and the sex that the gene is significant in are shown. Genes that are shared between the sexes are marked as ‘both’ and are in gray boxes. FDR values are shown for females and males. (XLSX 16 kb)

Additional file 3: **Table S3.** Gene rankings of sensitized PPI hubs in female and male data sets. (XLSX 836 kb)

Additional file 4: **Table S4.** Gene ontologies of the five focal regions described in Fig. 7. GO Process, GO function and GO component terms are shown for each region, along with their metrics. (XLSX 137 kb)

Additional file 5: **Table S5.** MtDNA-sensitive genes are often under-enriched in housekeeping gene lists. Hypergeometric analyses of enrichment of mtDNA sensitive genes (FDR < 0.05) among housekeeping gene lists are shown for three cluster types (Breadth, Tau and Small). Significant (*P* < 0.05) deviations from the expected overlap are highlighted in bold. (XLSX 13 kb)

Additional file 6: **Table S6.** Cluster Locator analyses of the FDR < 0.05 and top 200-ranked genes across four independent isogenic nuclear backgrounds and both sexes. Results are qualitatively similar in both analyses and both test sets are significantly clustered in three out of four nuclear backgrounds. For the top 200 gene set, all four nuclear backgrounds demonstrate significant clustering. (XLSX 12 kb)

Additional file 7: **Figure S1.** WGCNA-MtDNA-sensitive genes are found in low abundance in ‘proper’ modules. Eight proper gene modules of co-expressed genes, as revealed by WGCNA and represented by different colors are shown in A to H. Nodes represent genes and grey lines are edges connecting genes. Large nodes have a *p*-value < 0.05, while large nodes with red outer rings are significant at FDR < 0.05. Three contrast types are shown: ΔmtDNA ‘haplotype’, ΔmtDNA ‘species’, and G x G. The majority of significant genes are found in the improper (grey) module (not shown), with zero statistical support for module membership. (PDF 608 kb)

Additional file 8: **Figure S2.** Mapping DE gene enrichments using SAFE and a PPI network [49] analysis (all female genotype contrasts). Enriched regions of DE genes in the PPI network are shown as a heat component. Red hotspots show enriched regions of DE genes corresponding with: ΔmtDNA ‘haplotype’ (A), ΔmtDNA ‘species’ (B), G x G ‘mitonuclear epistasis’ (C), *Zim53-OreR* (D), *Zim53-si*I (E), *Zim53-sm21* (F), *OreR-si*I (G), *OreR-sm21* (H), and *si*I*-sm21* (I). Contrasts show both conserved and private regions of enrichment across contrasts. (PDF 1117 kb)

Additional file 9: **Figure S3.** Top ranked DE genes are physically clustered in the genome; a pattern consistently observed across independent nuclear genetic backgrounds and sexes. Physical clustering erodes with increasing p-value in the ΔmtDNA DE analysis. Each plot shows the window position of non-overlapping 200 gene groups from a ranked-by-p-value DE gene list. The most significant 200 genes are on the far left of each plot (window 1) and increasing p-value genes are associated with higher value 200-gene windows. The ordinal scale shows the significance (−log10 p-value) of the statistical clustering obtained using the cluster locator package as in the main analysis. Results from four independent nuclear backgrounds are shown: DGRP-315 (A, B); DGRP-820 (C, D); OregonR (E, F); and AustriaW132 (G, H). Females are shown in (A, C, E, G); males are shown in (B, D, F, H). A local weighted regression curve is shown in red in each plot. (PDF 5 kb)

Additional file 10: **Figure S4.** *Giant* has the strongest transcription factor enrichment score for mtDNA effects across four nuclear backgrounds and two sexes. The TF with the lowest rank (median TFBS enrichment rank: ordinal axis) has consistently the highest z-score; a measure of TFBS enrichment. The median rank across eight nuclear backgrounds x sex combinations is shown in blue for each transcription factor. Individual data are plotted in empty black circles. Transcription factors are ranked on the abscissa by their increasing median rank. (PDF 10 kb)

## Data Availability

Raw RNA-seq reads generated in this study are available from the NCBI Sequence Read Archive (SRA) (https://www.ncbi.nlm.nih.gov/sra) under project accession: PRJNA515519. Fly strains (and haplotype genetic sources) are available upon request.

## Abbreviations

*DGRP*: Drosophila Genetic Reference Panel
*DroID*: Drosophila Interactions Database
*G x G x E*: gene-by-gene-by-environment interaction
*G x G*: gene-by-gene interaction (epistasis)
*mtDNA*: mitochondrial DNA
*UCSC*: University of California Santa Cruz
*WGCNA*: Weighted Gene Co-expression Network Analysis
