# Supplementary figures and images for: Mitochondrial genomic variation drives differential nuclear gene expression in discrete regions of Drosophila gene and protein interaction networks
Source: BMC Genomics. 2019 Sep 2;20:691. doi: 10.1186/s12864-019-6061-y (PMC6719383; doi:10.1186/s12864-019-6061-y)

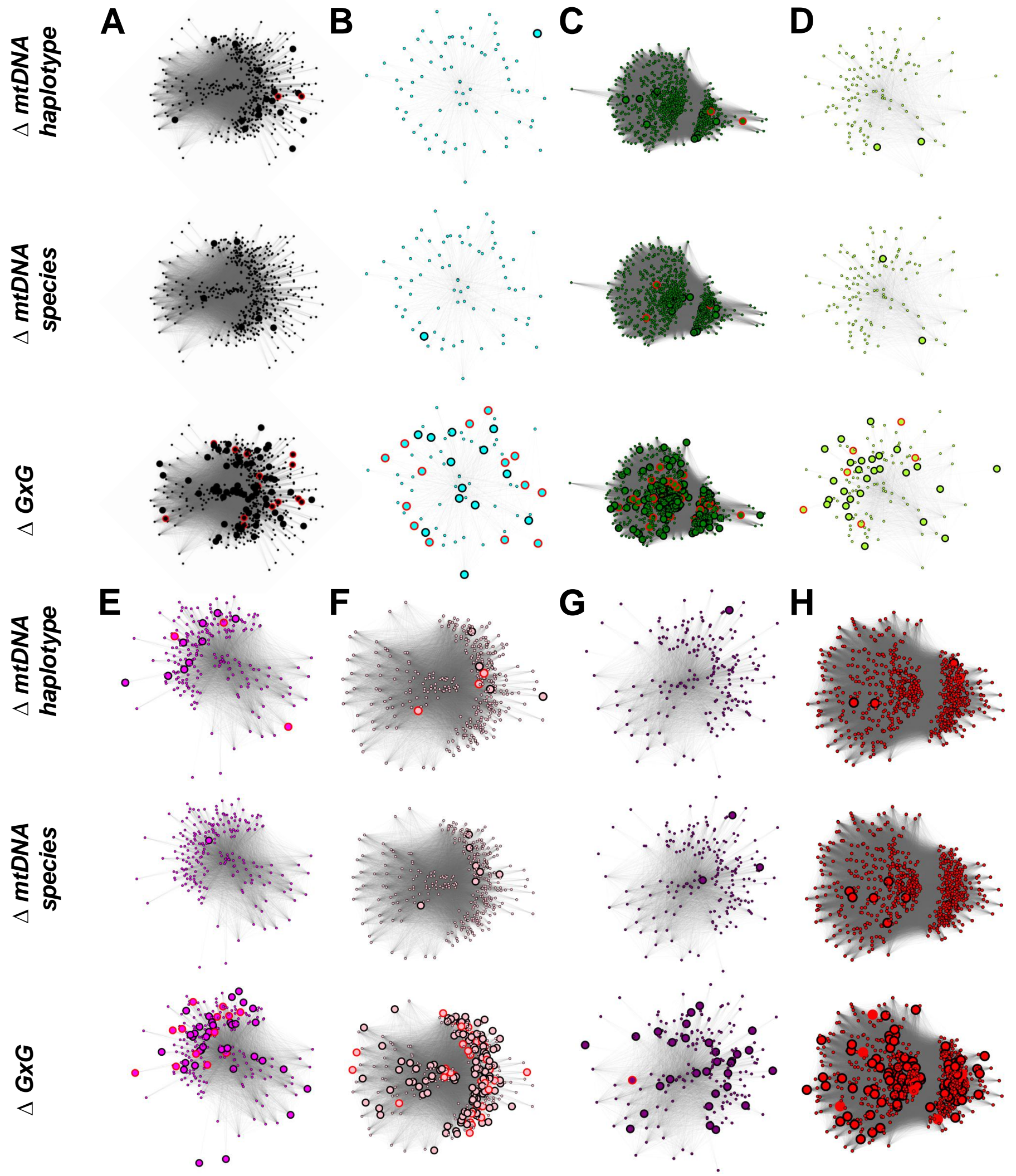

Supplement: Supplementary file 7 — Figure S1. WGCNA-MtDNA-sensitive genes are found in low abundance in ‘proper’ modules. Eight proper gene modules of co-expressed genes, as revealed by WGCNA and represented by different colors are shown in A to H. Nodes represent genes and grey lines are edges connecting genes. Large nodes have a p-value < 0.05, while large nodes with red outer rings are significant at FDR < 0.05. Three contrast types are shown: ΔmtDNA ‘haplotype’, ΔmtDNA ‘species’, and G x G. The majority of significant genes are found in the improper (grey) module (not shown), with zero statistical support for module membership. (PDF 608 kb) [file 12864_2019_6061_MOESM7_ESM.pdf]

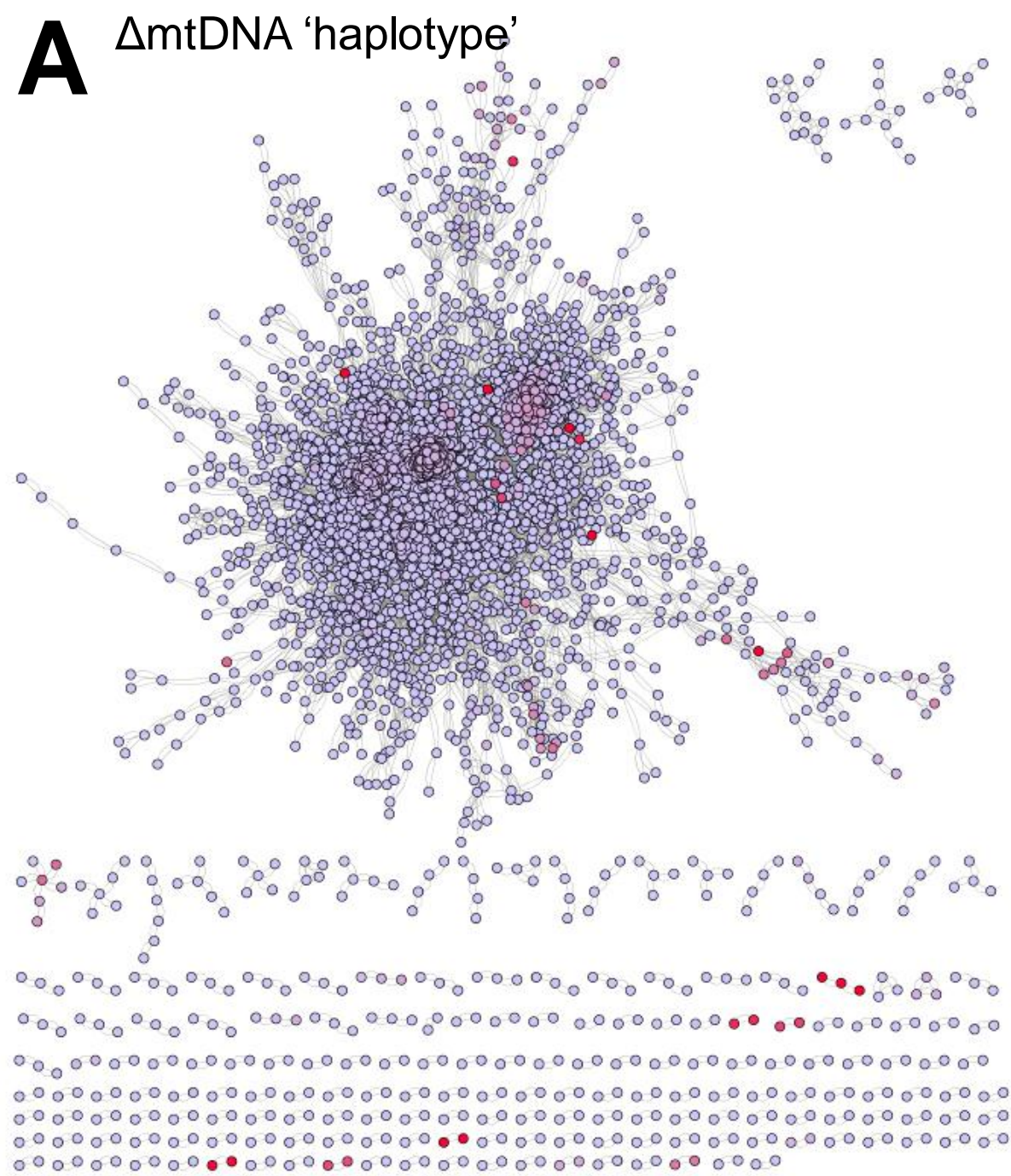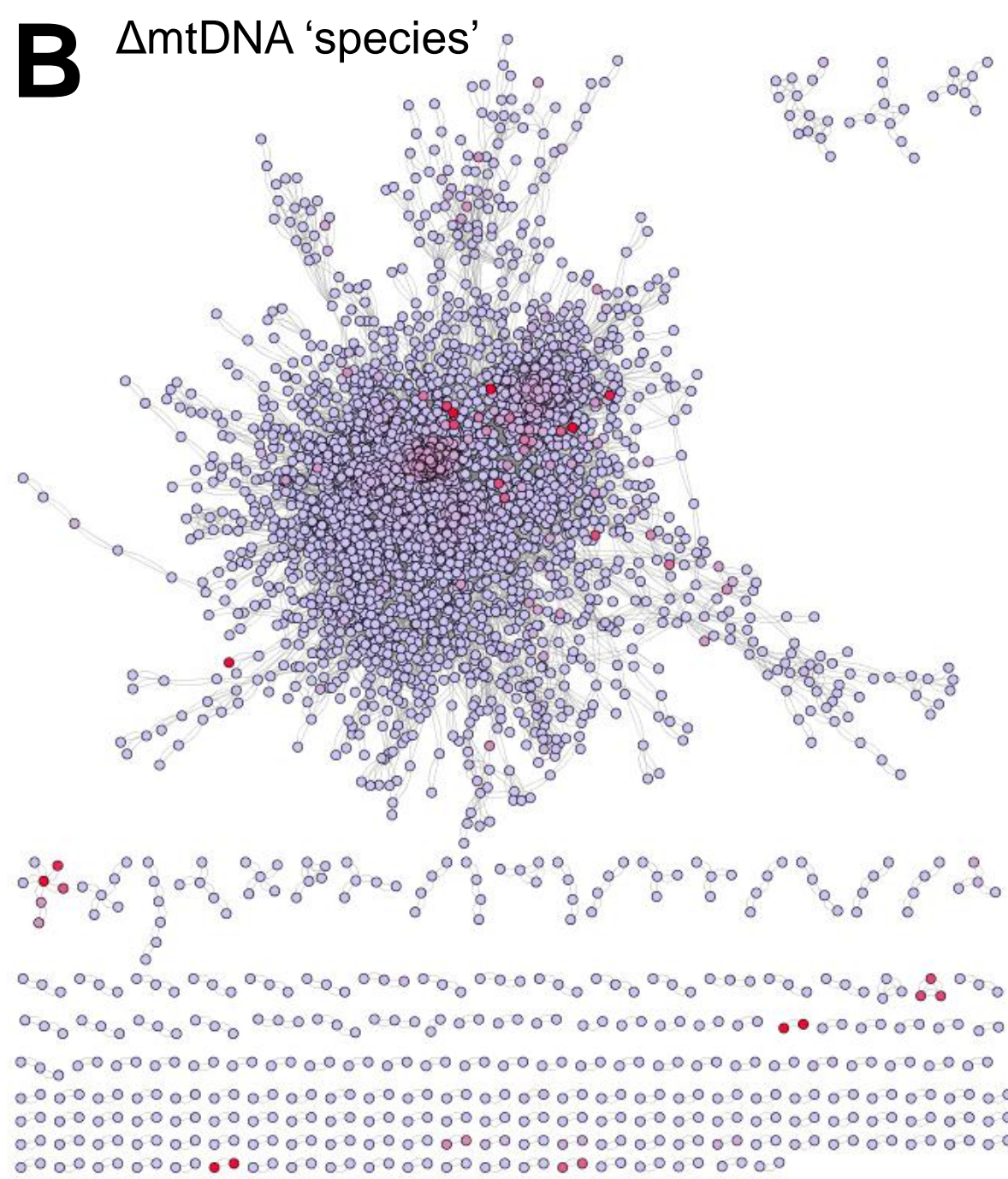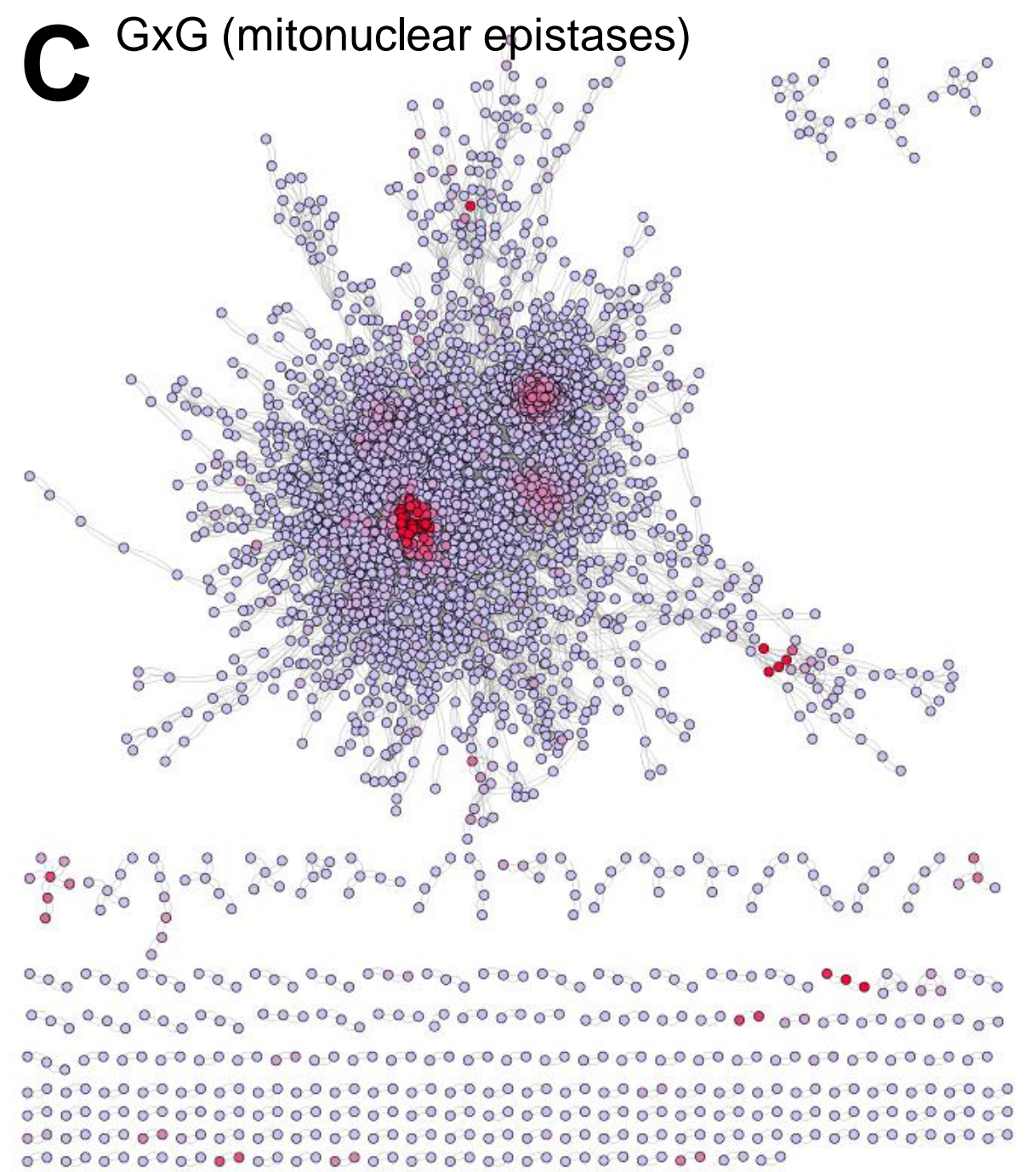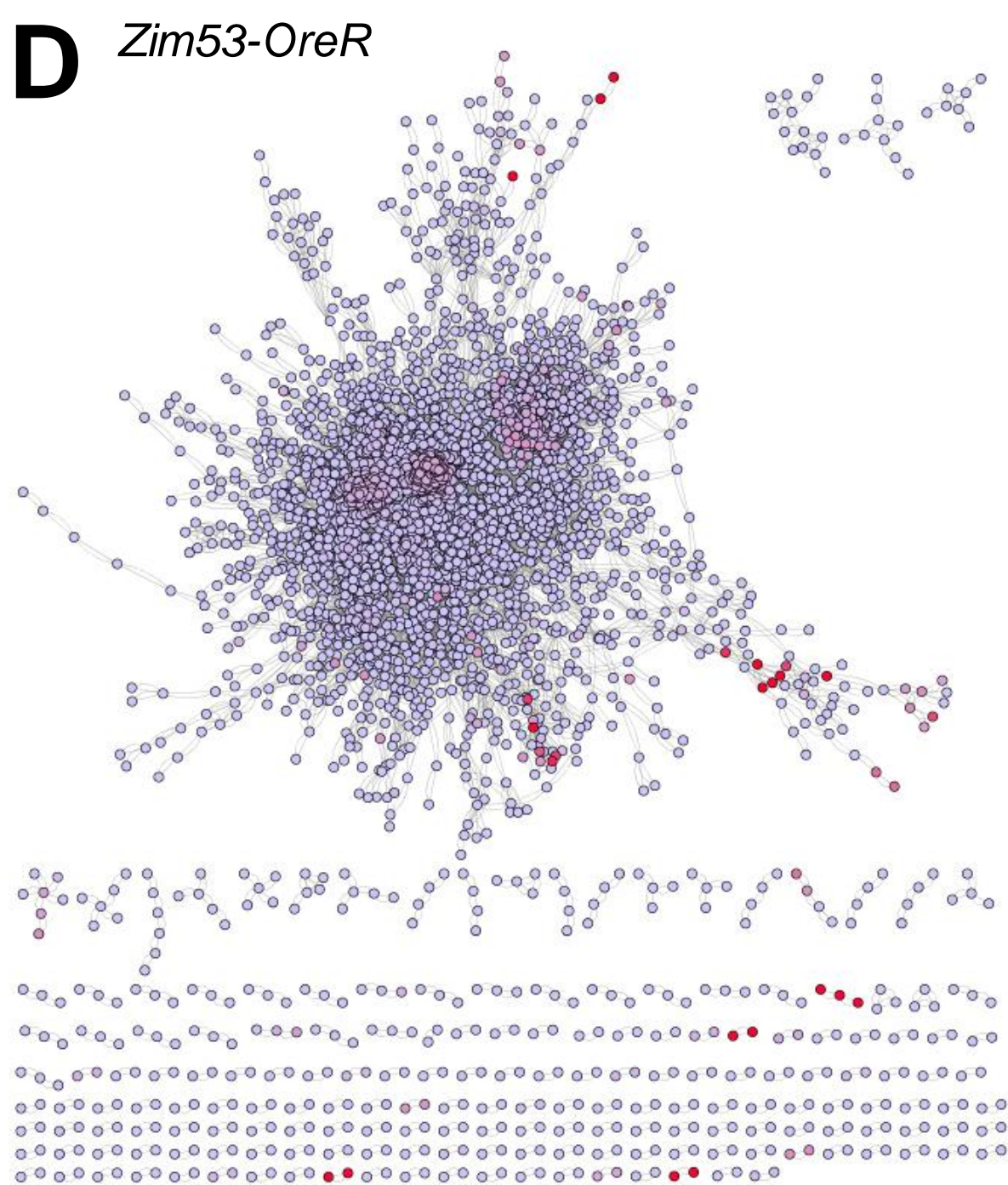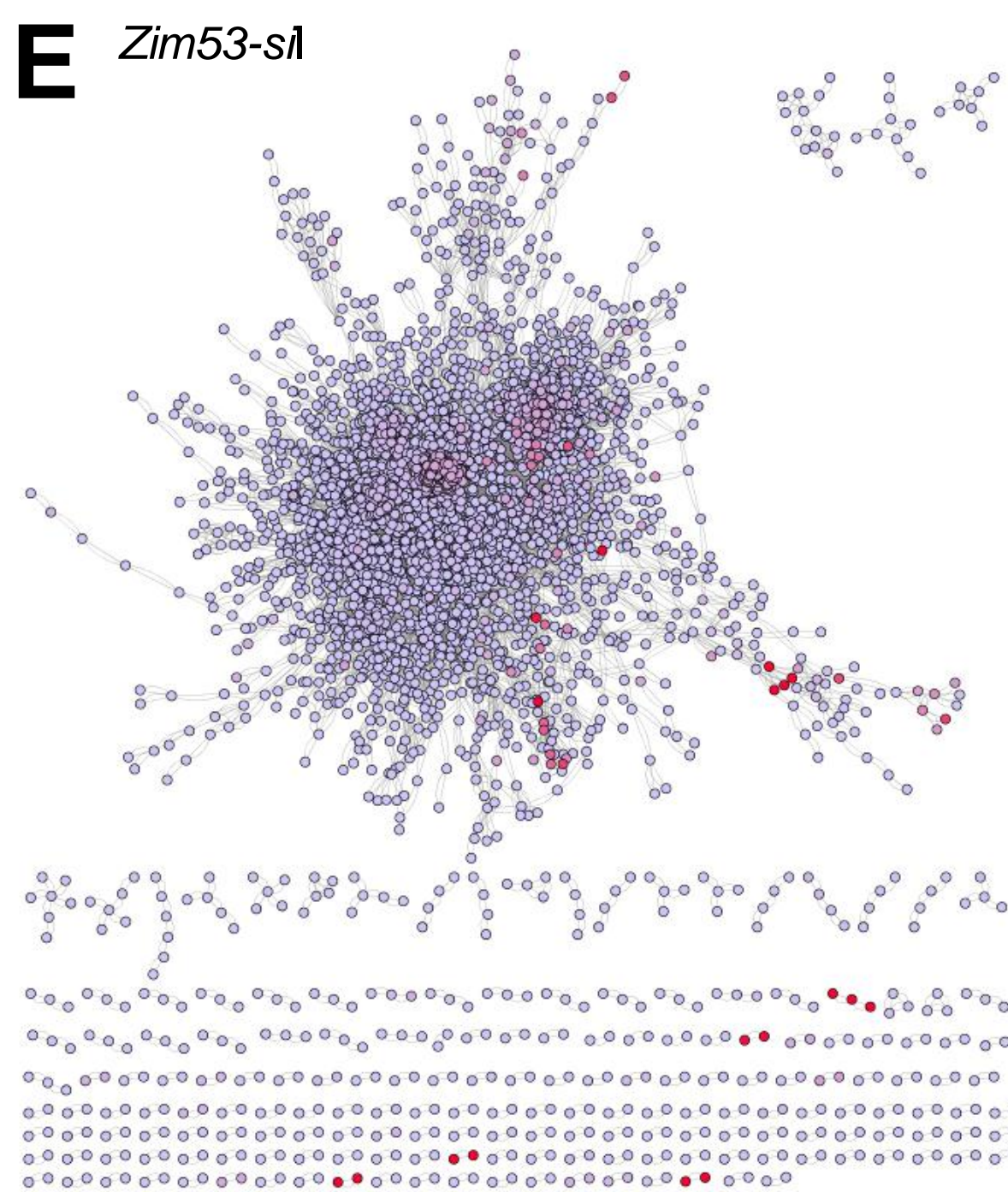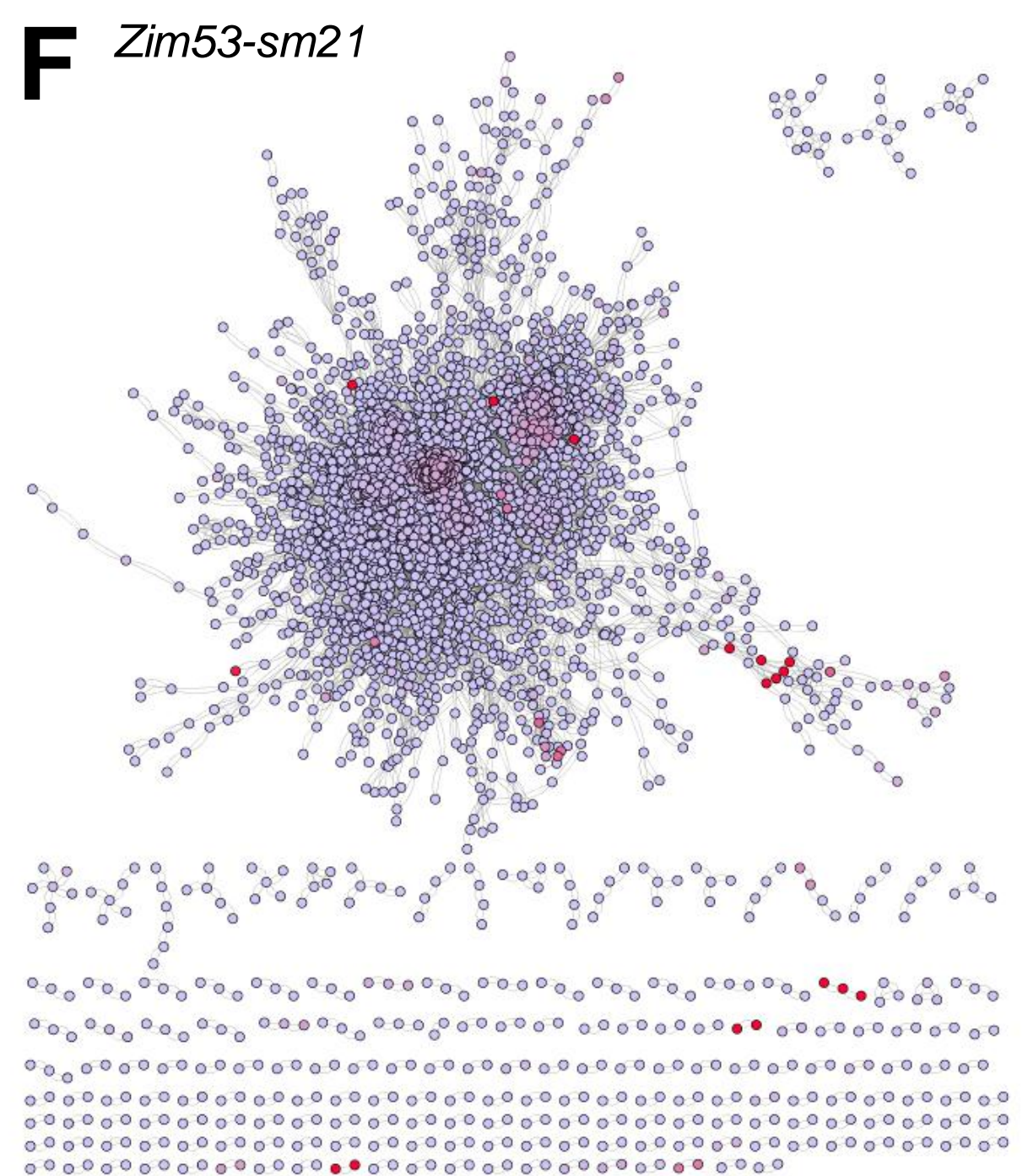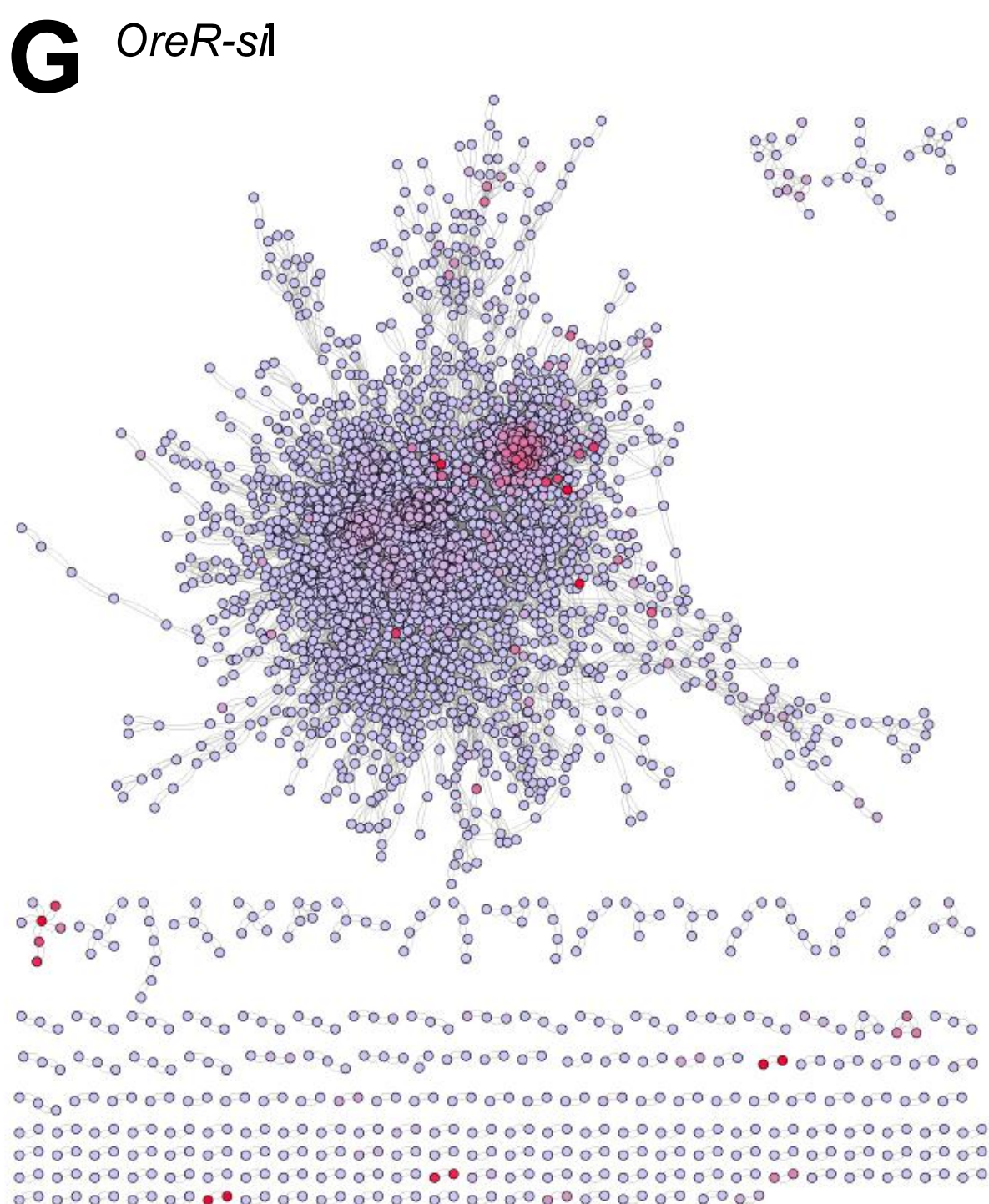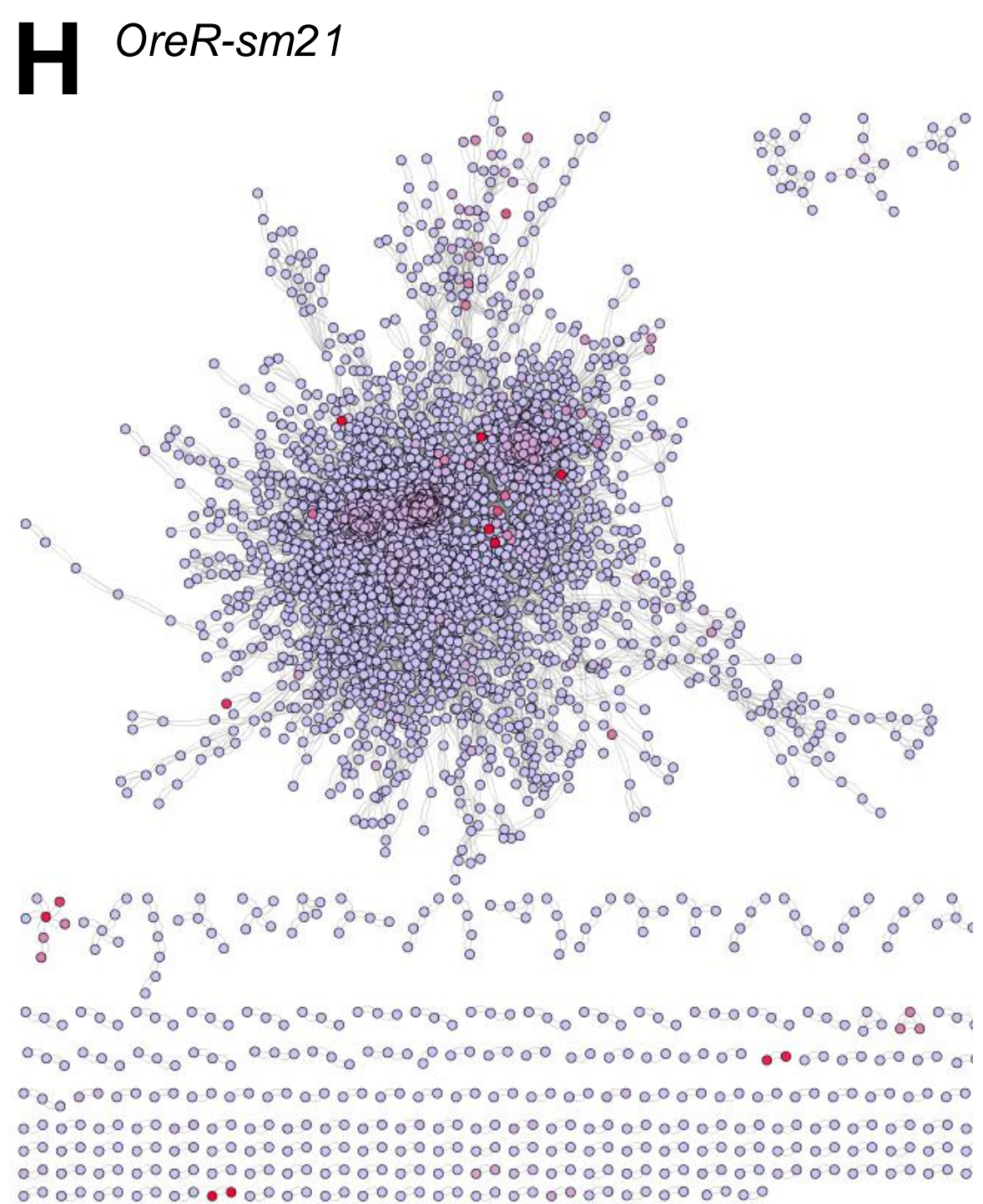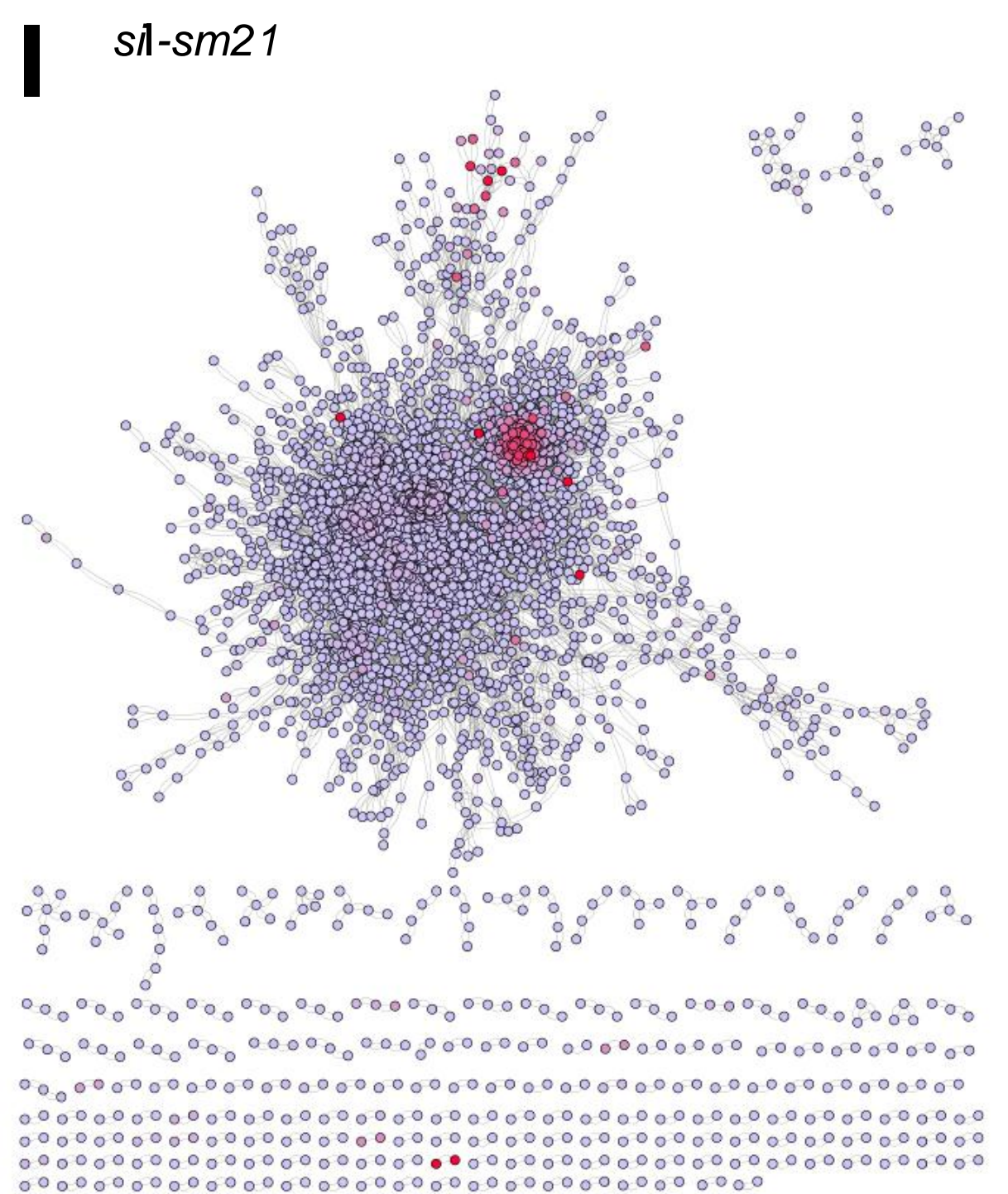

SAFE enrichment

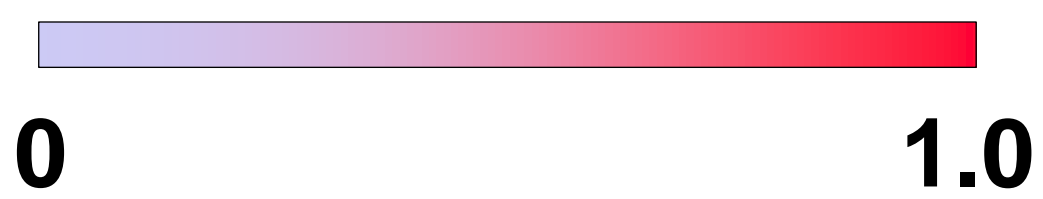

Supplement: Supplementary file 8 — Figure S2. Mapping DE gene enrichments using SAFE and a PPI network [49] analysis (all female genotype contrasts). Enriched regions of DE genes in the PPI network are shown as a heat component. Red hotspots show enriched regions of DE genes corresponding with: ΔmtDNA ‘haplotype’ (A), ΔmtDNA ‘species’ (B), G x G ‘mitonuclear epistasis’ (C), Zim53-OreR (D), Zim53-siI (E), Zim53-sm21 (F), OreR-siI (G), OreR-sm21 (H), and siI-sm21 (I). Contrasts show both conserved and private regions of enrichment across contrasts. (PDF 1117 kb) [file 12864_2019_6061_MOESM8_ESM.pdf]

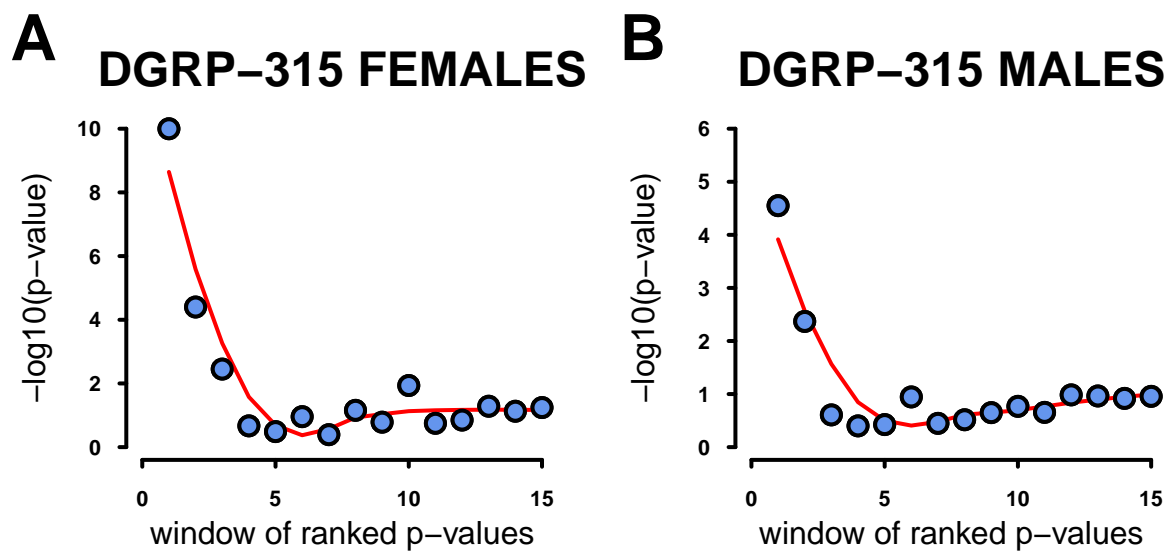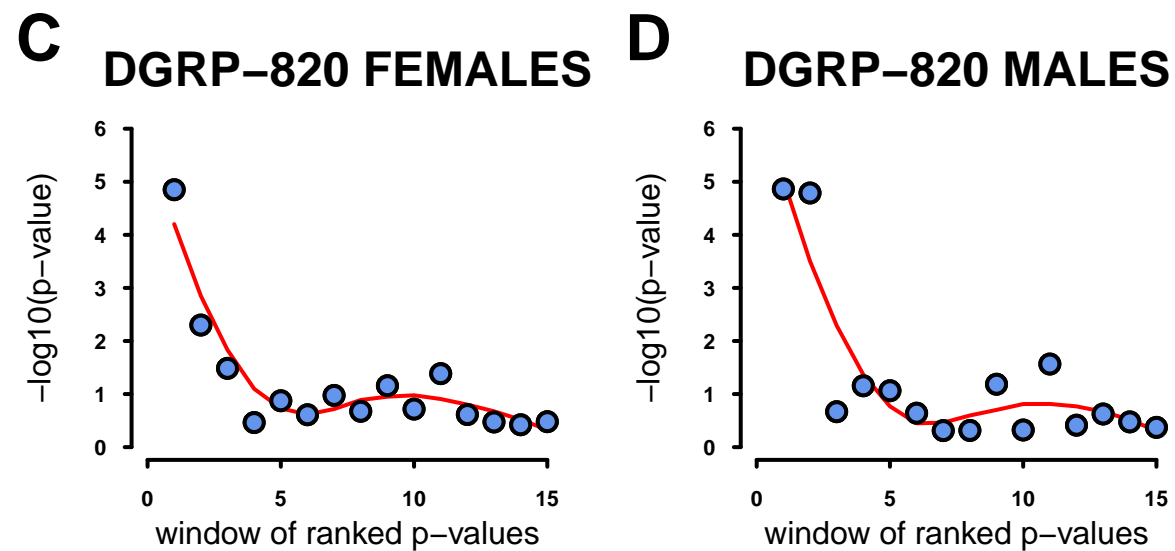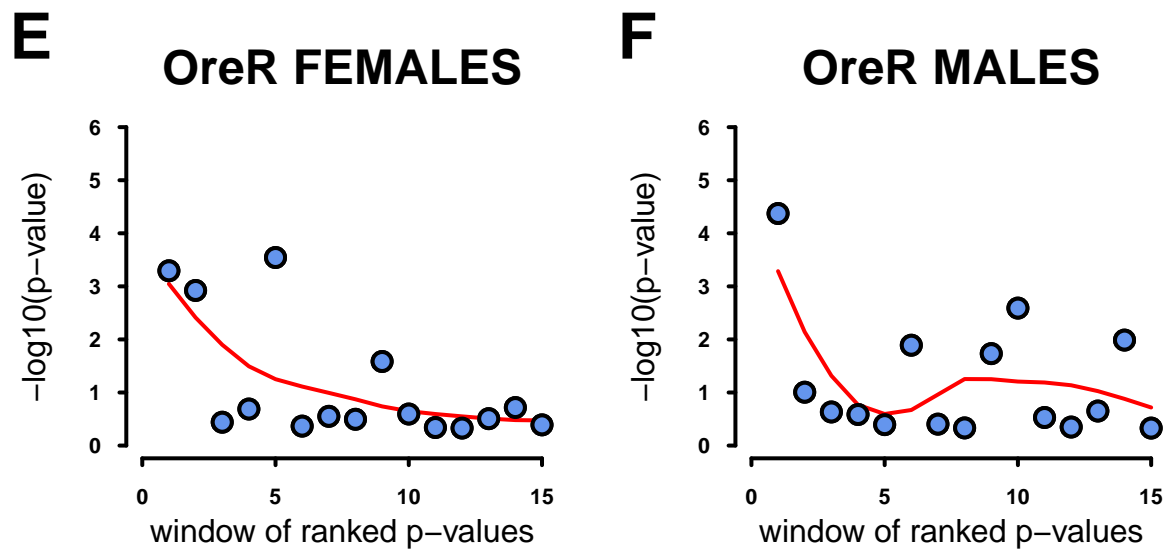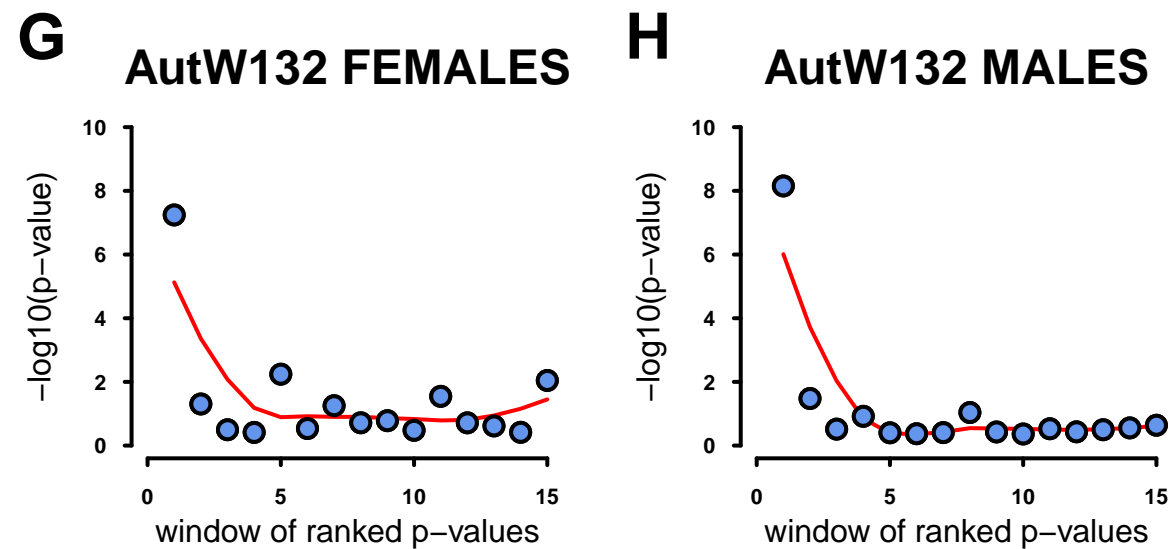

Supplement: Supplementary file 9 — Figure S3. Top ranked DE genes are physically clustered in the genome; a pattern consistently observed across independent nuclear genetic backgrounds and sexes. Physical clustering erodes with increasing p-value in the ΔmtDNA DE analysis. Each plot shows the window position of non-overlapping 200 gene groups from a ranked-by-p-value DE gene list. The most significant 200 genes are on the far left of each plot (window 1) and increasing p-value genes are associated with higher value 200-gene windows. The ordinal scale shows the significance (−log10 p-value) of the statistical clustering obtained using the cluster locator package as in the main analysis. Results from four independent nuclear backgrounds are shown: DGRP-315 (A, B); DGRP-820 (C, D); OregonR (E, F); and AustriaW132 (G, H). Females are shown in (A, C, E, G); males are shown in (B, D, F, H). A local weighted regression curve is shown in red in each plot. (PDF 5 kb) [file 12864_2019_6061_MOESM9_ESM.pdf]

TFBS enrichments across four independent nuclear backgrounds and both sexes

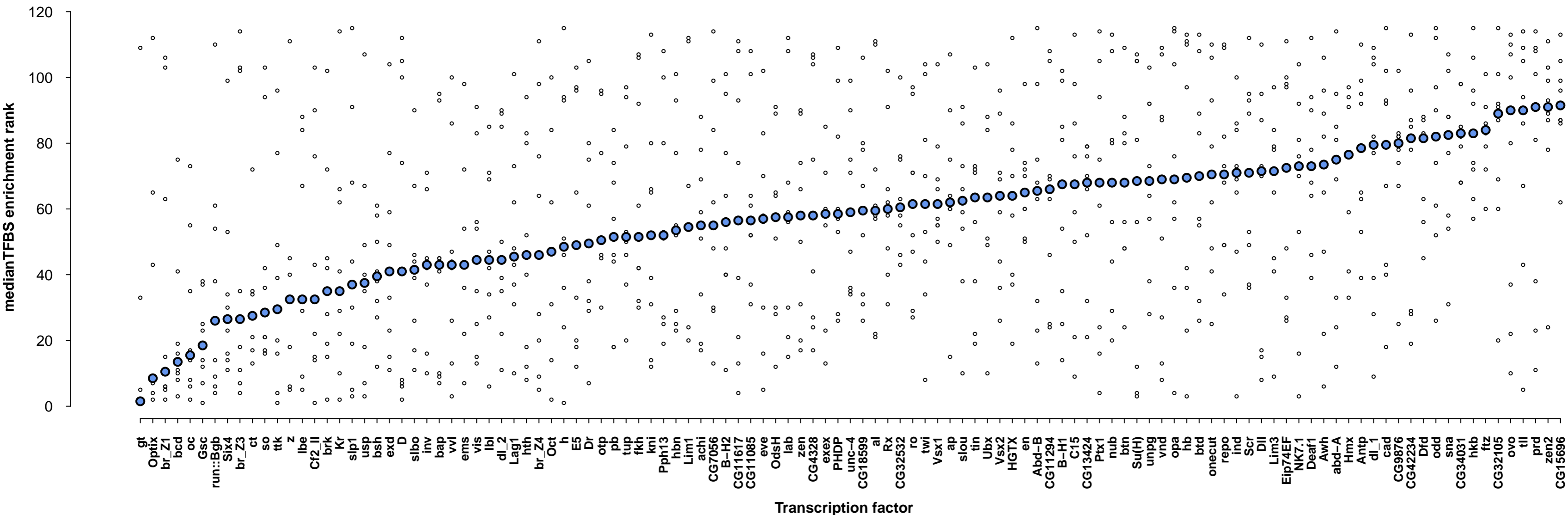

Supplement: Supplementary file 10 — Figure S4. Giant has the strongest transcription factor enrichment score for mtDNA effects across four nuclear backgrounds and two sexes. The TF with the lowest rank (median TFBS enrichment rank: ordinal axis) has consistently the highest z-score; a measure of TFBS enrichment. The median rank across eight nuclear backgrounds x sex combinations is shown in blue for each transcription factor. Individual data are plotted in empty black circles. Transcription factors are ranked on the abscissa by their increasing median rank. (PDF 10 kb) [file 12864_2019_6061_MOESM10_ESM.pdf]
